# Differential Regulation of Phytoene Synthase PSY1 During Fruit Carotenogenesis in Cultivated and Wild Tomato Species (*Solanum* section Lycopersicon)

**DOI:** 10.3390/plants9091169

**Published:** 2020-09-09

**Authors:** Gleb I. Efremov, Maria A. Slugina, Anna V. Shchennikova, Elena Z. Kochieva

**Affiliations:** Institute of Bioengineering, Research Center of Biotechnology, Russian Academy of Sciences, 119071 Moscow, Russia; gleb_efremov@mail.ru (G.I.E.); shchennikova@yandex.ru (A.V.S.); ekochieva@yandex.ru (E.Z.K.)

**Keywords:** carotenogenesis, fruit color, *Solanum* section Lycopersicon, phytoene synthase 1

## Abstract

In plants, carotenoids define fruit pigmentation and are involved in the processes of photo-oxidative stress defense and phytohormone production; a key enzyme responsible for carotene synthesis in fruit is phytoene synthase 1 (PSY1). Tomatoes (*Solanum* section Lycopersicon) comprise cultivated (*Solanum lycopersicum*) as well as wild species with different fruit color and are a good model to study carotenogenesis in fleshy fruit. In this study, we identified homologous *PSY1* genes in five *Solanum* section Lycopersicon species, including domesticated red-fruited *S. lycopersicum* and wild yellow-fruited *S. cheesmaniae* and green-fruited *S. chilense*, *S. habrochaites* and *S. pennellii*. PSY1 homologs had a highly conserved structure, including key motifs in the active and catalytic sites, suggesting that PSY1 enzymatic function is similar in green-fruited wild tomato species and preserved in red-fruited *S. lycopersicum*. *PSY1* mRNA expression directly correlated with carotenoid content in ripe fruit of the analyzed tomato species, indicating differential transcriptional regulation. Analysis of the *PSY1* promoter and 5′-UTR sequence revealed over 30 regulatory elements involved in response to light, abiotic stresses, plant hormones, and parasites, suggesting that the regulation of *PSY1* expression may affect the processes of fruit senescence, seed maturation and dormancy, and pathogen resistance. The revealed differences between green-fruited and red-fruited *Solanum* species in the structure of the *PSY1* promoter/5′-UTR, such as the acquisition of ethylene-responsive element by *S. lycopersicum*, could reflect the effects of domestication on the transcriptional mechanisms regulating *PSY1* expression, including induction of carotenogenesis during fruit ripening, which would contribute to red coloration in mature fruit.

## 1. Introduction

Tomato (*Solanum lycopersicum* L.) fruit represents a fleshy berry, which during ripening, changes the color from green to red (most common) or yellow, orange, pink, brown, or purple, primarily because of chlorophyll degradation and intensification of carotenoid synthesis and accumulation [1,2]. *S. lycopersicum* belongs to the *Solanum* section Lycopersicon, which also includes 12 related wild tomato species with different evolutionary ages. Most wild tomatoes produce green or dark-green fruit, with the exception of three species, which have yellow (*Solanum cheesmaniae* [L. Riley] Fosberg and *Solanum galapagense* S.C. Darwin and Peralta) or red (*Solanum pimpinellifolium* B. Juss.) fruit color depending on the lycopene to β-carotene ratio [3,4]. All tomato species have flower petals of bright yellow-orange color due to the accumulation of carotenoids neoxanthin, violaxanthin, and lutein [5]. Carotenoids not only color flower organs and fruit to attract insects and animals for pollination and seed dispersion but are also involved in photosynthesis, photo-oxidative stress defense, and phytohormone production during plant vegetative growth [6,7,8,9,10].

The biosynthesis of carotenoids, including the underlying genetic and enzymatic networks, is described in many plant species [11]. The carotenoid synthetic pathway is initiated in plastids by phytoene synthase (PSY), which is present in plastid stroma (chloroplasts, chromoplasts and amyloplasts) and catalyzes the condensation of two geranylgeranyl diphosphate (GGPP) molecules to yield 15-*cis*-phytoene [2,12]. The following consecutive biosynthetic reactions catalyzed by phytoene desaturase (PDS), ζ-carotene isomerase (ZISO) and ζ-carotene desaturase (ZDS) produce cis-lycopene, which is then converted by carotenoid isomerase (CrtISO) into trans-lycopene (red pigment) further used for the synthesis of α- and β-carotenes (orange pigments), which in turn may be converted to lutein and xanthophylls, respectively [1,2,13]. In this pathway, PSY is the key enzyme, and its loss blocks carotenoid synthesis during fruit ripening, whereas its overexpression increases carotenogenesis in sink organs in several crop species, including potato and tomato [14,15].

Plants may have from one to four PSY-encoding genes [16,17,18]. In tomato, two active PSY enzymes were initially described: PSY1 (pTOM5) and PSY2, which provide carotenoid synthesis in fruit and photosynthetic tissues, respectively; both of them are necessary for petal pigmentation [12,19,20]. Paralogous *PSY1* and *PSY2* genes, which are considered to emerge as a result of the *Solanum*-specific whole-genome triplication and subsequent subfunctionalization [12,21], encode proteins with highly homologous structures; however, their biochemical properties are slightly different [12,22]. Thus, the carotenogenic activity of PSY1 is lower than that of PSY2, which may be the reason behind a higher level of *PSY1* gene transcription during carotenoid synthesis in ripe fruit [10]. The fact that all tomato species have the same color of flower petals (yellow) but differ in the color of ripe fruit depending on the evolutionary origin (ancient species have green and recent species—yellow-to-red fruit) may indicate that *PSY1* emerged later than *PSY2* and was initially involved, together with *PSY2*, in flower coloration and only later acquired a distinct function in fruit pigmentation [5]. The recently identified third tomato PSY enzyme (PSY3) mainly functions in the synthesis of root apo-carotenoids (C_19_ strigolactones, C_15_ abscisic acid, and C_13_/C_14_ apo-carotenoids) involved in establishing plant symbiotic and parasitic relationships and adaptation to nutrient deficiency [23]. *PSY3* homologs have been found in both monocots [24,25,26] and eudicots [23,27,28] up to *Amborella trichopoda* Baill., the most basal angiosperm considered a progenitor of extant angiosperms [23,29], suggesting an earlier origin of the root-specific *PSY3* gene compared to the fruit-specific *PSY1* gene in tomato.

Tomato species (*Solanum* section Lycopersicon) are considered a good model to study fleshy fruit ripening, including coloration and evolution of the underlying genetic mechanisms, because of their significant phenotypic diversity [30]. All three *PSY* genes have been detected in cultivated tomato *S. lycopersicum*; however, there are few studies on PSY enzymes in wild tomatoes, which mostly indicate that fruit color depends on PSY1 expression [31,32,33,34,35].

The aim of this study was to evaluate the interspecific variability of *PSY1* homologous genes in cultivated and wild tomato accessions, reveal possible associations of PSY1 structural polymorphisms and expression with carotenoid biosynthesis and fruit color, and determine PSY1 relevance to the evolution of tomato species. 

## 2. Results

### 2.1. Identification and Structural Analysis of PSY1 Homologous Genes in Solanum Section Lycopersicon Species

**Figure 1:**
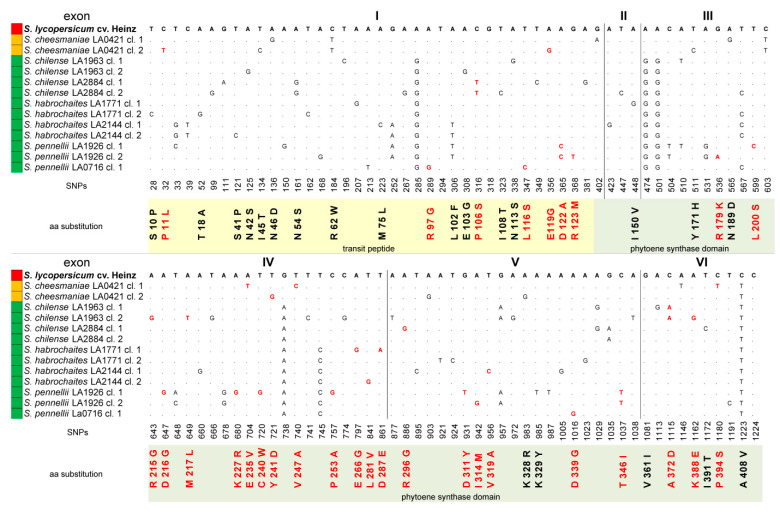
Polymorphisms in identified tomato PSY1 homologs. The numbers indicate the positions of single nucleotide polymorphisms (SNPs) in the *PSY1* coding sequences (exons I–VI) relative to *S. lycopersicum PSY1*; the resulting amino acid substitutions in the translated PSY1 proteins are shown below. Non-synonymous SNPs and PROVEAN-predicted radical amino acid substitutions are marked red. Color of the ripe fruit is indicated to the left of the species name.

A total of 13 complete sequences of *PSY1* homologous genes, including their allelic variants, were amplified from genomic DNA of eight accessions of five tomato species: red-fruited (RF) *S. lycopersicum*, yellow-fruited (YF) *S. cheesmaniae* and green-fruited (GF) *S. chilense*, *S. habrochaites* and *S. pennellii.* The genes were cloned and sequenced, and the data deposited in the NCBI GenBank (accession numbers are shown in Table 1). The size of the identified *PSY1* genes ranged from 4840 bp (*S. chilense* LA1963 clone 2) to 4916 bp (*S. habrochaites* LA1771 clone 2) (Table 1); all genes included 5′-UTR (~1.5 kb), a promoter (~0.9 kb) and a coding (3.3 kb in average from the start to stop codon) sequence. The open reading frame (ORF) was 1239-bp long and contained six exons; the length and number of exons, and, consequently, the cDNA size did not vary among the analyzed species and were the same as reported for *S. lycopersicum PSY1* [12] and other *Solanum PSY1* genes available in the NCBI database (Table 1, Figure 1).

Compared to the *PSY1* gene of *S. lycopersicum* cv. Heinz 1706, the average variability of the identified *PSY1* genes was 12.8% for complete sequences (641 SNPs in the aligned 4995-bp gene portion): 8.5% for exons (105 SNPs in 1239 bp), 12.2% for introns (298 SNPs in 2117 bp), and 14.6% for 5′-UTR (238 SNPs in 1639 bp); 5′-UTR and exon I were the most variable parts. In total, 51 (48.57%) exonic SNPs were non-synonymous and found mostly in *PSY1* homologs of GF tomatoes (Figure 1).

The same analysis performed for other *Solanum* species indicated that the variability of their *PSY1* genes was mostly lower than that in the *Solanum* section Lycopersicon species. Thus, in potato (*Solanum* section Petota), it was only 8.00% (414 SNPs in 5150 bp), including 5.2% for exons and 6.98% for 5′-UTR, and in more distant *Solanum* species (*S. sisymbriifolium*, *S. melongena*, *S. prinophyllum*, and *S. torvum*), it was 6.2%, 6.4%, 5.8% and 8.5% for exons, respectively. Other members of the Solanaceae family, such as *Capsicum annuum*, *Lycium* species, *Nicotiana tabacum*, and *Petunia axillaris* had 7.9%, 9.0%, 11.3% and 16.1% variability, respectively, in *PSY1* genes compared to *PSY1* of *S. lycopersicum* cv. Heinz 1706. 

### 2.2. Structural Analysis of Tomato PSY1 Homologs

The size of the translated PSY1 proteins was the same for all studied tomato species: 412 amino acids (aa) (Table 1). Similar to *S. lycopersicum* PSY1, the putative PSY1 protein homologs contained a conserved phytoene synthase domain head-to-head (HH)-IPPS (75–405 aa, according to the NCBI-CDD) characteristic for the isoprenoid biosynthesis C1 superfamily. The N-terminal transit peptide (TP) was determined according to a previous study [12]; its predicted cleavage site V61/R62 was the same for the analyzed PSY1 homologs, excluding V61/W62 in PSY1 of *S. cheesmaniae* LA0421. However, according to Resource UniProtKB (Mitofates, Predotar, and TargetP tools) the PSY1 phytoene synthase domain was mapped to 130–412 aa and TP—to 1–129 aa, which is consistent with the plastidic localization of PSY1; in this case, the TP cleavage site was located at A129/E130 and was the same for all analyzed PSY1 proteins.

Compared to the PSY1 of cultivated *S. lycopersicum* (cv. Heinz 1706), the analyzed PSY1 homologs from wild tomatoes had 46 residue substitutions (11.2% of the 412-aa protein), of which 27 (58.7%) were radical (Figure 1). The 62-aa TP encoded by exon I was the most variable PSY1 region, containing seven substitutions (15.2%), of which only one was predicted to be radical (rP11L in *S. cheesmaniae* clone 2). In case of the 129-aa TP, 18 substitutions (39.1%) were found; among them, seven were predicted to be radical, of which four were located close to the cleavage site (Figure 1). Most of the radical replacements were detected in *S. pennellii* LA0716 clone 1 (rR97G, rP106S, rL116S, and rD122A); they were also found in *S. chilense* LA2884 clones 1 and 2 (rP106S), *S. cheesmaniae* LA0421 clone 2 (rE119G), and *S. pennellii* LA1926 clone 2 (rD122A and rR123M) (Figure 1). The remaining 28 substitutions (including 20 radical) were located in the conserved HH-IPPS domain. The HH-IPPS domains of two RF species, *S. pimpinellifolium* and *S. lycopersicum*, differed from each other only by one neutral substitution (nV360I); *S. pimpinellifolium* PSY1 lacked nA408V found in all analyzed accessions (Figure 1). PSY1 of YF *S. cheesmaniae* LA 0421 contained seven aa substitutions (nN189D, rE235V, rV247A, and rP394S in clone 1 and nY171H, rY241D and nK328R in clone 2) (Figure 1).

The majority of residue substitutions (19, including 16 radical) were found in the PSY1 HH-IPPS domain of more ancient GF tomato species. Almost all substitutions were species- and accession-specific; they were found in *S. chilense* LA1963 (rR215G, rM217L, rK388E and rA372D in clone 2 and rA372D in clone 1) and LA2884 (rR296G and nI391T in clone 1), *S. habrochaites* LA1771 (nI150V, rE266G, and nD287E in clone 1) and LA2144 (rL281V in clone 2), and *S. pennellii* LA1926 (rL200S, rD216G, rK227R, rC240W, rD311Y and rT346I in clone 1; rT346I, rR179K and rI314M in clone 2) and LA0716 (rD339G in clone 1) (Figure 1). Thus, among the analyzed tomato species, *S. pennellii* LA1926 PSY1 contained the most variable HH-IPPS domain and *S. pennellii* LA0716 PSY1—the most polymorphic TP, whereas minimal differences were observed between the PSY1 homologs of RF and YF species.

Polymorphisms in PSY1 homologs of species from *Solanum* sections Petota and Melongena, *S. prinophyllum* and *S. torvum*, as well as other Solanaceae members *C. annuum*, *Lycium* species, *N. tabacum*, and *P. axillaris* constituted 4.6%, 5,6%, 6.2%, 7.7%, 8.1%, 11.0%, 6.8% and 14.4%, respectively, compared to *S. lycopersicum* cv. Heinz 1706 PSY1.

**Figure 2:**
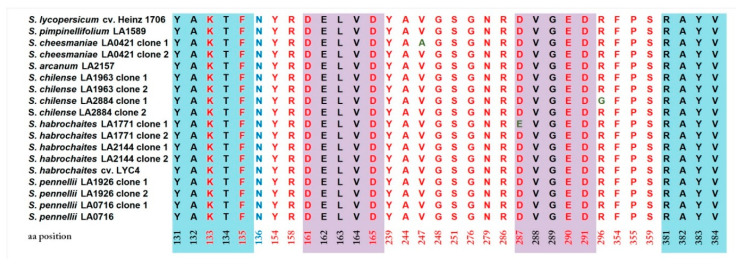
Functionally important sites in *PSY1*-encoded proteins. Active site residues (131-YAKTF-135 and 381-RAYV384) are highlighted in blue, aspartate-enriched substrate-Mg^2+^ binding sites (161-DELVD-165 and 287-DVGED-291)—violet, and additional residues that form the active site are in red font. Amino acid positions are indicated according to *S. lycopersicum* cv. Heinz PSY1. Position N136 (in blue font) indicates the Y136N replacement compared to *C. annuum* PSY1.

To expand the data on the variability of functionally important sites among tomato species, an expanded list of tomato PSY1 homologs (supplemented with PSY1 proteins available from NCBI, in particular, from *S. pimpinellifolium* and *S. arcanum*) was analyzed using the NCBI Conserved Domains Database, and the active site lid residues (131-YAKTF-135 and 381-RAYV-384; define the conformation of the active cleft site), aspartate-rich substrate-Mg^2+^ binding sites (161-DELVD-165 and 287-DVGED-291), substrate-binding pockets (K133, F135, Y154, R158, D161, D165, Y239, A244, G248, G276, N279, R286, D287, E290, D291, R296, F354, and P355), and catalytic residues (F135, Y154, D161, D165, Y239, V247, S251, N279, R286, D287, D291, R296, F354, P355, and S359) were revealed (Figure 2). The identified functionally important sites were conserved among the analyzed PSY1 proteins, with the exception of substitutions in the second substrate-Mg^2+^ binding site (nD287E in *S. habrochaites* LA1771 clone 1) and the substrate-binding/catalytic site (rR296G in *S. chilense* LA2884 clone 1). Furthermore, all identified tomato PSY1 homologs contained a functionally important mutation Y136N absent in other Solanaceae species (Figure 2).

**Figure 3:**
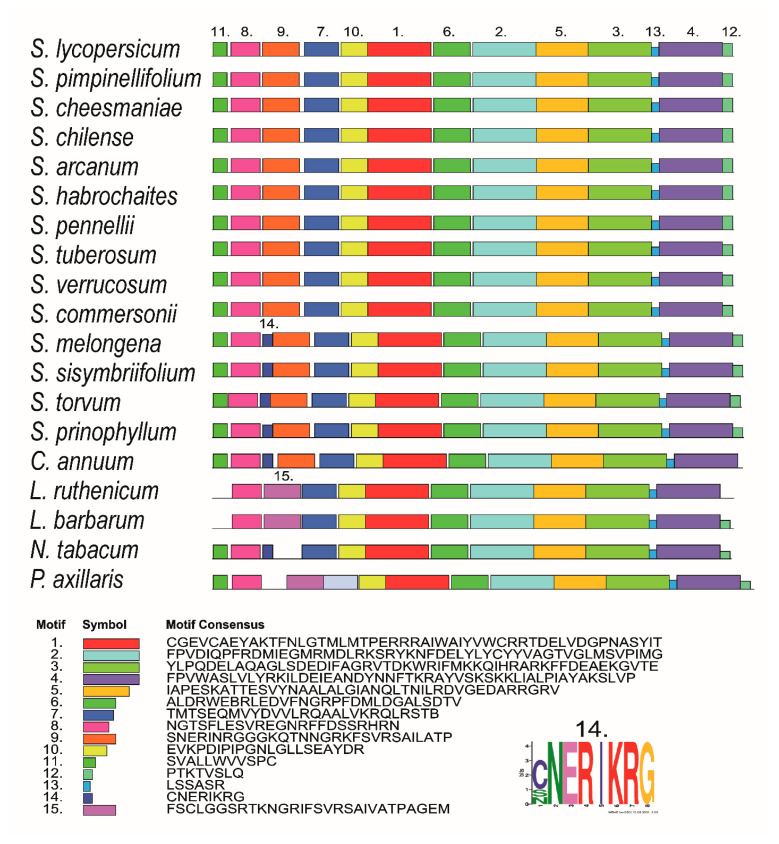
Distribution of conserved motifs in PSY1 homologs of Solanaceae species identified using the MEME search tool. The length of each box corresponds to that of the motif; the order of the motifs corresponds to their position in each protein.

MEME-based analysis revealed 13 conserved motifs in PSY1 proteins of the analyzed tomato accessions and potato species. Additional group-specific motifs were found in *S. melongena*, *S. sisymbriifolium*, *S. torvum*, *S. prinophyllum*, and *C. annuum* (motif 14; 41–48 aa), and in *Lycium* and *Petunia* species (motif 15; 42–70 and 60–88 aa, respectively) (Figure 3).

### 2.3. Spatial Structure of Tomato PSY1 Homologs

The three-dimensional (3D) structure of tomato PSY1 homologs was predicted based on crystal structures of four related enzymes: the C(30) carotenoid dehydrosqualene synthase 2 from *Staphylococcus aureus* (25–26% identity; PDB: 3W7F), dehydrosqualene synthase (29–31%; 5IYS), squalene synthase HpnC from *Alicyclobacillus acidocaldarius* (27–29%; 4HD1), and putative phytoene/squalene synthase YisP from *Bacillus subtilis* (26–27%; 3WE9). The results indicated that similar to the structures of reference enzymes, that of tomato PSY1 was rich in anti-parallel α-helices, which formed a large central cavity making the catalytic site.

Overall, we could model 288–300 aa-long fragments representing 69–71% of the PSY1 protein (including the 109–408-aa region with the HH-IPPS domain) with more than 90% confidence. Similar results (284–300-aa; 68–73%) were obtained for PSY1 homologs from *S. tuberosum*, *S. commersonii*, *S. verrucosum* (sect. Petota), *L. ruthenicum*, *L. chinense*, *L. barbarum*, *S. torvum*, *S. sisymbriifolium*, *S. melongena*, *S. prinophyllum*, *C. annuum*, *N. tabacum*, *P. axillaris*, and *A. thaliana*. The remaining 27–32% residues were modeled *ab initio*.

**Figure 4:**
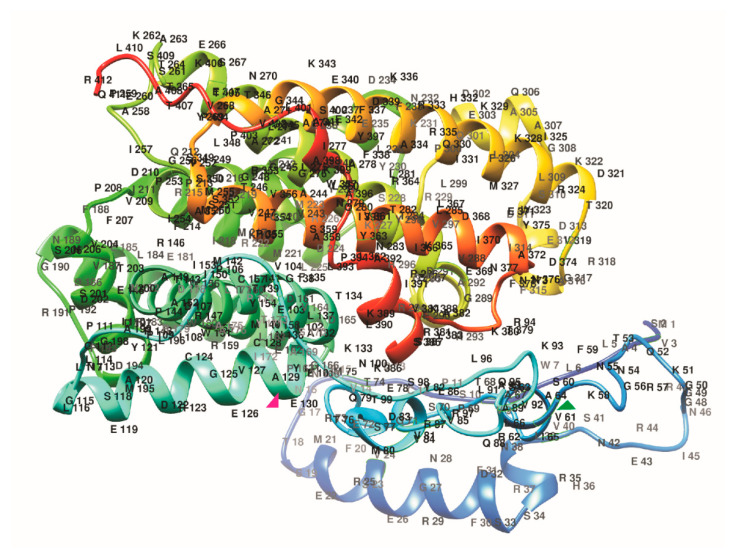
Spatial structure of *S. lycopersicum* cv. Heinz 1706 PSY1 built using Phyre2. Chains are colored according to the rainbow spectrum from the N- to C-terminus. Two residues predicted to flank TP (V61 and A129) are indicated by green and pink triangles, respectively.

No species or fruit color-specific structural features were observed in TP or the HH-IPPS domain. Considering that reliable modeling covered the 114–401-aa region of PSY1, which included a domain conserved in dehydrosqualene synthases (also belonging to the isoprenoid biosynthesis C1 superfamily), the obtained data may support the predicted 1–129-aa location of TP in PSY1, which could extend as far as the end of the fourth α-helix (L117–E129), i.e., up to the active site (130-YAKTF-134) (Figure 4). Although the TP sequence varied among plant PSY1 enzymes, its C-terminus (α-helix with motif LSEAYDRCGEVCAE) was modeled with more than 90% confidence, suggesting its conservation as the region preceding the cleavage site.

### 2.4. PSY1-Based Phylogeny of Tomato Species 

**Figure 5:**
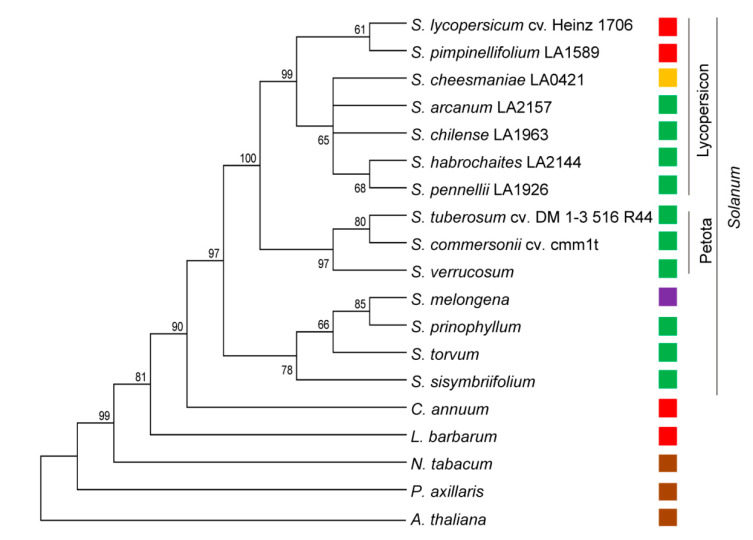
Evolutionary relationship among Solanaceae species (Solanum lycopersicum, Solanum pimpinellifolium, Solanum cheesmaniae, Solanum arcanum, Solanum chilense, Solanum habrochaites, Solanum pennellii, Solanum tuberosum, Solanum commersonii, Solanum verrucosum, Solanum melongena, Solanum prinophyllum, Solanum torvum, Solanum sisymbriifolium, Capsicum annuum, Lycium barbarum, Nicotiana tabacum, and Petunia axillaris) based on the PSY1 coding sequences. Arabidopsis thaliana (Brassicaceae) PSY was used as an outgroup. Analysis was performed using the neighbor-joining method. The optimal tree with the sum of branch length = 2.35980228 is shown. Percentages of replicate trees in which the associated taxa clustered together in the bootstrap test (1000 replicates) are shown next to the branches. The tree is drawn to scale, with branch lengths in the same units as those of the evolutionary distances used to infer the phylogenetic tree. The evolutionary distances were computed using the Jukes–Cantor method and are in the units of the number of base substitutions per site. For each species, the color of the ripe fruit is indicated to the right of the name.

The coding sequences of the identified and already known *PSY1* genes were used to analyze the phylogeny of the Solanaceae family. The foundation of the Solanaceae phylogenetic tree was occupied by *P. axillaris*, followed by *N. tabacum*, *L. barbarum*, and *C. annuum* (Figure 5). The *Solanum* species were the most recent; eggplant and the related species were grouped in the basic sub-cluster, whereas sister clades of the Petota and Lycopersicon species were positioned at the top of the tree (Figure 5). Tomatoes were divided into two main clades containing RF and GF species, respectively. The GF clade was rooted by the most ancient species *S. habrochaites* and *S. pennellii* grouped together, and included YF *S. cheesmaniae* (Figure 5). 

### 2.5. PSY1 Expression Pattern

*PSY1* expression was tested in young leaves, flower buds with green petals, yellow petals, and mature green (MG) and ripe fruit of four tomato species differing in fruit color—GF *S. habrochaites* LA2144 and *S. pennellii* LA0716, YF *S. cheesmaniae* LA0421, and RF *S. lycopersicum* (cv. Heinz 1706-BG). The aim was to analyze a possible correlation between the level of *PSY1* expression and the content of carotenoids in fruits, and the choice of species, in addition to the ripe fruit color, was determined by the species phylogeny: RF *S. lycopersicum* as the most recent species, YF *S. cheesmaniae* as an evolutionary older species, and also the two most ancient GF species *S. pennellii* and *S. habrochaites*. *S. chilense* was not included in the comparative analysis; this species is another GF tomato, evolutionary located between the recent RF and YF and the most ancient GF species, and its fruits contained the same amount of carotenoids as other GF species.

**Figure 6:**
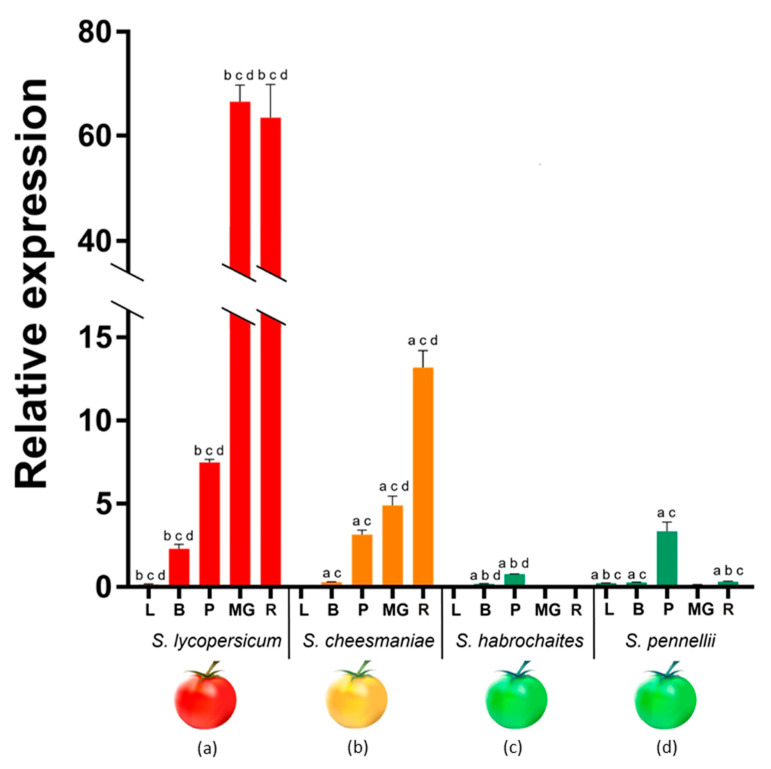
*PSY1* mRNA expression in leaves (L), young flower buds (B), yellow petals (P), and mature green (MG), and ripe (R) fruit of *S. lycopersicum* cv. Heinz 1706, *S. cheesmaniae* LA0421, *S. habrochaites* LA2144, and *S. pennellii* LA0716. Low-case letters above the bars indicate statistically significant differences (*p*-value < 0.005) between gene expression levels in the same tissue of different species: *S. lycopersicum* (a), *S. cheesmaniae* (b), *S. habrochaites* (c), and *S. pennellii* (d). For example, *PSY1* expression in *S. lycopersicum* leaves differed significantly from those in *S. cheesmaniae*, *S. habrochaites* and *S. pennellii* leaves, which is, respectively, denoted by three letters “bcd” above the L-bar.

In YF *S. cheesmaniae* and RF *S. lycopersicum*, *PSY1* mRNA was expressed in all tested tissues, except for the leaves of *S. cheesmaniae*; *PSY1* expression was consistently upregulated from a very low level in leaves to a high level in ripe fruit in both species; however, it was about five times higher in *S. lycopersicum* than in the *S. cheesmaniae* fruit (Figure 6).

In GF *S. pennellii*, *PSY1* transcription was also detected in all tested tissues; however, in contrast to YF and RF species, it was maximal in yellow petals and minimal in MG and ripe fruit, where its level was approximately 5–13 times lower than in *S. cheesmaniae*, and 65 times lower than in *S. lycopersicum*. In another GF species *S. habrochaites*, *PSY1* mRNA was detected, at a very low level, only in buds and petals (Figure 6).

*PSY1* expression was minimal or absent in the leaves of all four tomato species and very low in buds of all wild accessions. The highest *PSY1* mRNA level in yellow petals was observed in *S. lycopersicum*, where it exceeded that in *S. cheesmaniae*/*S. pennellii* and *S. habrochaites* by two and 10 times, respectively (Figure 6).

### 2.6. Carotenoid and Chlorophyll Content

**Figure 7:**
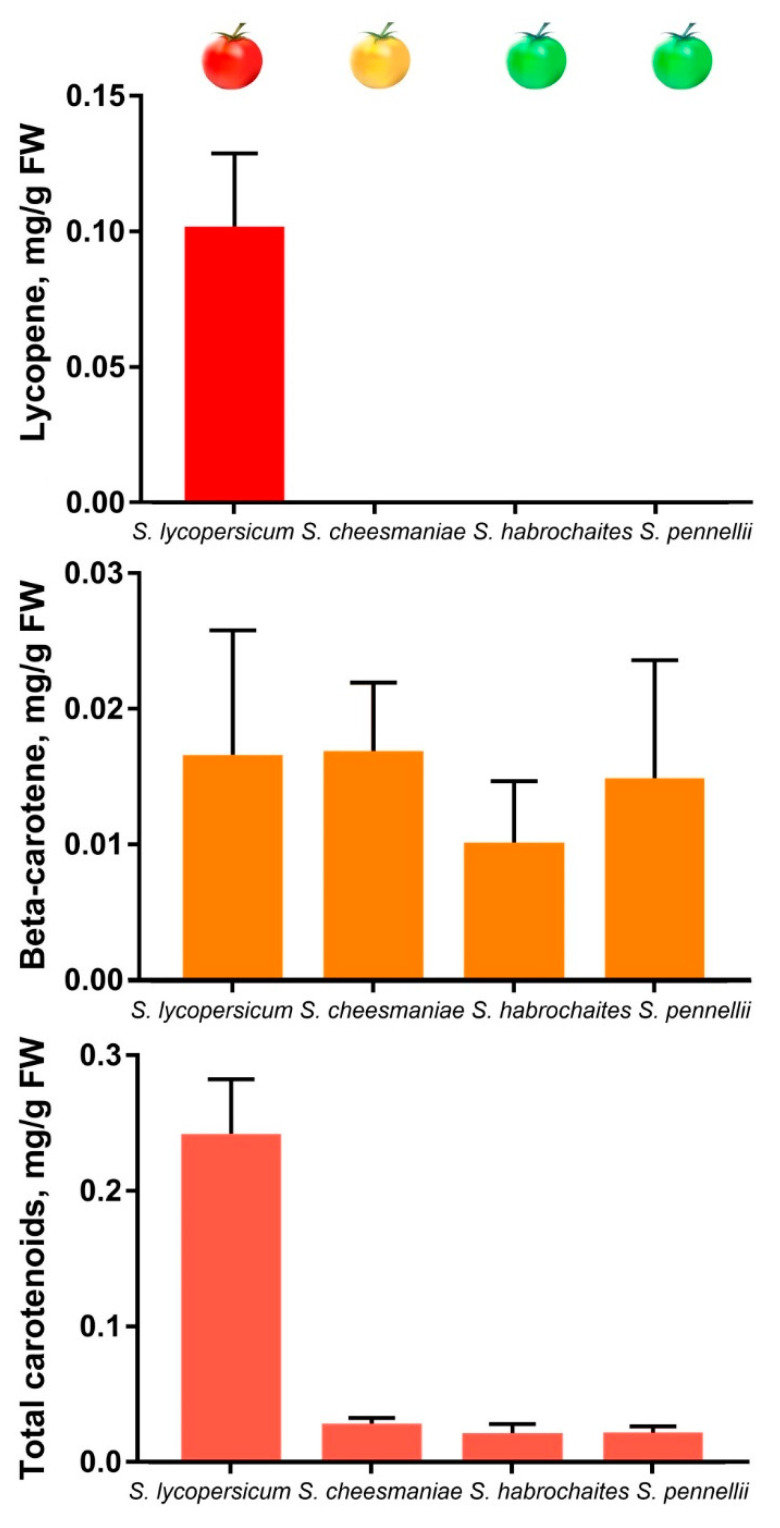
Carotenoid content in ripe fruit of tomato species *S. lycopersicum* cv. Heinz 1706, *S. cheesmaniae* LA0421, *S. habrochaites* LA2144, and *S. pennellii* LA0716.

Accumulation of total (x + c) and specific carotenoids (lycopene and β-carotene), as well as colorless carotenoid precursors (phytoene and phytofluene) and chlorophylls *a* and *b*, was assessed in ripe fruit and leaf of GF species *S. habrochaites* (LA2144), *S. pennellii* (LA0716) and *S. chilense* (LA1963), YF *S. cheesmaniae* (LA0421) and RF *S. lycopersicum* (cv. Heinz 1706-BG). The results indicated that the fruit of wild tomatoes lacked lycopene and contained 10–20 times less of total carotenoids than those of *S. lycopersicum*; however, fruit β-carotene content was the same for all analyzed species (Table 2, Figure 7). At the same time, the content of total carotenoids in fruit was significantly lower than in leaf (Table 2). However, leaf-specific carotenogenesis is mainly mediated by another phytoene synthase isoform PSY2; thus, a comparison of the carotenoid content in fruit and leaf could be correct if the ripe green fruit consisted only of photosynthetic tissues, which is obviously not the case. Chlorophyll content in the fruit of GF tomatoes was considerably reduced compared to the leaves, whereas in the fruit of YF *S. cheesmaniae* and RF *S. lycopersicum* it was absent at all (Table 2).

In the fruit of GF species, the content of total carotenoids was almost equal to the β-carotene content (Figure 7, Table 2), which means the lowest possible amount of other types of colorful carotenoids. In the fruit of YF *S. cheesmaniae*, β-carotene accounted for about 2/3 of the total carotenoids (Figure 7, Table 2), which may indicate the accumulation of α-carotene and lutein, as was previously shown for *yellow-fruited tomato 1* mutant fruit [37]. We assume that the fruit of *S. cheesmaniae* produce so little lycopene that it could be fully converted into α- and β-carotene by lycopene cyclases. 

The potential accumulation of phytoene and phytofluene was assessed by the absorption spectra of fruit extracts. The characteristic spectral features of colorless carotenoid precursors were revealed only in the red ripe fruit of *S. lycopersicum* cv. Heinz, while in the spectra of ripe fruit of GF and YF accessions, these spectral features were not recorded, which indicates a minor content of carotenoid precursors (Supplementary File).

### 2.7. Promoter and 5′-UTR Analysis

We next analyzed the promoter and 5′-UTR of the *PSY1* gene in two tomato species, RF *S. lycopersicum* cv. Heinz 1706 and it’s most distant wild relative GF *S. pennellii* LA0716. A comparison of the identified *S. lycopersicum* sequences with those from the NCBI database (*S. lycopersicum* cv. Heinz 1706; *PSY1* Gene ID: 543988, NC_015440.3 [4350836…4355976]) revealed no differences, whereas in the identified *S. pennellii* LA0716 *PSY1*, 10 nucleotides inserted and 31 SNPs were detected compared to *S. pennellii* LA0716 *PSY1* (Gene ID: CCXL01009615.1 [3669…9559]).

The sizes of the promoter/5′-UTR were 906/1535 bp in *S. lycopersicum* and 919/1550 bp in *S. pennellii*. Compared to the *S. lycopersicum PSY1* promoter and 5′-UTR, the corresponding *S. pennellii PSY1* regions contained 56 and 63 SNPs, indicating 6% and 3.98% variability, respectively; these variability levels were significantly higher than those in the whole gene (from start to stop codon), cDNA and protein: 2.54%, 0.8% and 0.95% (86, 10 and 3 substitutions), respectively.

Search for sites important for *PSY1* transcription revealed that the *PSY1* promoter and 5′-UTR contained 37 types of regulatory elements related to tissue-specific expression and response to phytohormones and stress factors. Among them, 32 were common for *PSY1* of both species, including core promoter elements and 11 were species-specific; among the latter, there were light- and ethylene-responsive elements, sites associated with reactions to salicylic acid, cold and wounding and binding sites of the MYB transcription factor implicated in drought response (Table 3). Compared to the ancient *S. pennellii*, the *S. lycopersicum PSY1* promoter/5′-UTR region lost light-(chs-Unit 1 m1 and TCT motif), salicylic acid-(two TCA sites) and drought-(MBS) responsive elements and an AAGAA motif with unknown function, while acquiring other light-responsive elements (3-AF3 binding site and GATA motif) as well as ethylene-(ERE) and wound-(WUN-motif) responsive elements and an AACCTAACCT motif with unknown function (Table 3).

## 3. Discussion

The existing wide variety of tomato cultivars, which differ in fruit color and other economically important traits, are derived from the most evolutionary recent species *S. lycopersicum*. It is proposed that tomato domestication began in South America, when wild RF species *S. pimpinellifolium* gave rise to *S. lycopersicum* var. *cerasiforme*, further evolution of which in Mesoamerica resulted in *S. lycopersicum* var. *lycopersicum* [38,39] that, together with its 12 wild relatives, now form *Solanum* section Lycopersicon [3].

Tomato diversification due to domestication is distinct from the natural species divergence as it passed through various genetic bottlenecks caused by the selection of a limited set of traits valuable for humans, which significantly limited genomic diversity among modern *S. lycopersicum* cultivars [36,40,41]. As a result, *S. lycopersicum* differs from its wild ancestors in a wide range of acquired morphophysiological characteristics (so-called domestication syndrome) and in genetic diversity, which constitutes no more than 5% of that existing among wild tomatoes [39]. Fruit color and carotenoid content are included in the domestication syndrome; these features are controlled by qualitative trait genes, including *PSY1* encoding phytoene synthase, the key enzyme of carotenogenesis in fruit, which has a conserved structure but also contains polymorphisms associated with the evolution of plant species [42,43].

In the present study, *PSY1* homologous genes were identified and characterized in one cultivated *(S. lycopersicum* cv. Heinz) and four wild (*S. pennellii*, *S. habrochaites*, *S. chilense*, and *S. cheesmaniae*) tomato species that differ in ripe fruit color (Table 1). All identified homologs were highly conserved and had the same functionally important residues, indicating that the enzymes should have the same catalytic mechanism underlain by the formation of the C-terminal catalytic site—a large central cavity composed of two anti-parallel alpha-helices and motifs DELVD and DVGED at the opposite sides of the cavity (Figure 2 and Figure 4) [44]. These data indicate that the catalytic function of the PSY1 homologs in carotenogenesis is conserved in wild and domesticated tomato species.

The N-terminal TP is responsible for PSY translocation into plastids [10,12], and its polymorphism may be one of the main species-specific signatures in plants [45]. Indeed, in the analyzed tomato species, the N-terminal TP was found to be the most polymorphic PSY1 region, which distinguished Lycopersicon species from the other Solanaceae members (Figure 3 and Figure 4). Furthermore, it appears that in the course of evolution, TPs of *Capsicum* and *Solanum* PSY1 enzymes have lost the FSCLGGSRTKNGRIFSVRSAIVATPAGEM motif and acquired the CNERIKRG motif instead, which was later deleted in Petota and Lycopersicon species (Figure 3).

It has been suggested that carotenogenic enzymes can be used to shed light on the molecular evolution of different plant species [43]. Phylogenetic analysis based on whole-genome sequence data or trait-encoding genes produces similar results, to which exons contribute more evolution-related information than introns as they are under higher selective pressure [42]. Therefore, in this study, we examined the possibility of using *PSY1* coding sequences to evaluate Solanaceae phylogeny (Figure 5). The results indicated that the analyzed tomato species were grouped according to the generally accepted evolution history of nightshades, which suggests major splits between tomato and potato (eight million years ago [Mya]), eggplant and tomato/potato (14 Mya), *Solanum* and *Capsicum* (19 Mya), and *Solanum* and *Nicotiana* (24 Mya) [46]. Fleshy fruit developed during the latter split had the ability to acquire color because of carotenogenesis. In this study, the analyzed tomatoes could be further divided into two main clades of RF and GF species based on the *PSY1* sequence (Figure 5), indicating that PSY1 may be a good phylogenetic marker to investigate the recent evolution of tomatoes.

In the GF clade, the most distant *S. habrochaites* and *S. pennellii* were grouped together, which is consistent with the incompletely resolved dichotomy between the two species belonging to different informal groups, Eriopersicon and Neolycopersicon, respectively [47]; sometimes, *S. pennellii* is considered to be an intermediate species between *S. habrochaites* and *S. arcanum* [42]. The other two informal groups are Lycopersicon, which includes all the YF-RF tomato species, and Arcanum [47]. Carotenoid accumulation is the main feature of Lycopersicon species: the red color of *S. lycopersicum* and *S. pimpinellifolium* fruit is caused by the accumulation of lycopene (up to 500-fold during ripening [40]), which in *S. cheesmaniae* and *S. galapagense* is further processed into β-carotene accounting for yellow-to-orange fruit color [48,49]. In Neolycopersicon, Eriopersicon, and Arcanum species, the level of carotenoid biosynthesis in fruit is similar to that in photosynthetic tissues [49].

Considering the conserved structure of PSY1 as the key carotenogenic enzyme in all tomato species, the question is why the fruit of *S. lycopersicum* turns red, whereas those of wild tomatoes remain green or only slightly yellow. The answer may lie in differential regulation of *PSY1* expression, the level of which positively correlates with carotenoid content. Thus, in maize, *PSY1* is transcribed in yellow endosperm but not in the carotenoid-deficient white endosperm [17,50] and in rice, *PSY1* overexpression makes white endosperm turn yellow because of β-carotene accumulation [51]. In RF *S. lycopersicum*, *PSY1* expression is low in photosynthetic tissues and high in petals and ripe fruit [11,12], whereas in GF species (*S. peruvianum*, *S. chilense*, *S. pennellii* and *S. chmielewskii*) *PSY1* is not expressed in ripe fruit, which do not accumulate lycopene and contain 100 times less β-carotene than those of RF tomatoes [31,32].

However, the question of why wild tomato ripe fruits remain green cannot be answered simply by the *PSY1* expression level. For instance, overexpression of *PSY* in *A. thaliana* does not turn photosynthetic leaves into orange or red, and the content of carotenoids there remains unchanged, while β-carotene accumulation in non-photosynthetic tissues sharply increases [52]. The green color of the ripe fruit in GF tomatoes may be due to the lack of special globular structures for carotenoid accumulation, which typically present in the chromoplasts of non-photosynthetic tissues, or to the impaired chromoplast formation [33,35,53,54]. In this regard, it is interesting that *PSY1* expression and PSY1 enzymatic activity are quite significant in the fruit of GF *S. habrochaites* (accession LA1777); however, there is no carotenoid accumulation because of impaired chromoplast formation [33] due to a mutation in the ORANGE protein [35,54].

Consistent with these observations, in the current study, we detected *PSY1* transcription in yellow-colored petals and red- and yellow-colored fruits but not in leaves (Figure 6), which confirms chromoplast specificity of *PSY1* expression in tomato species [12].

The absence of yellow-to-red pigments in the ripe green fruit may result from a metabolic bottleneck and blockage of reactions following PSY1-catalyzed phytoene biosynthesis. Then the ripe green fruit could accumulate an excess of colorless precursors, which in RF tomatoes are rapidly processed to form colorful carotenoids. In this study, significant amounts of phytoene and phytofluene were observed in the ripe fruit of only RF species (and these amounts directly correlated with the content of colorful carotenoids), but not in the GF or YF tomatoes (Supplementary file). Apparently, there is no blocking of the reactions following the synthesis of phytoene and phytofluene, and the low content of colorful carotenoids in the ripe fruit of GF and YF tomatoes may be explained by low amounts of carotenoid precursors. Thus, the final content of carotenoids in the ripe fruit is largely controlled by the level of *PSY1* expression, determining the amount of colorless precursors.

Overall, in this study, the content of colorful carotenoids in ripe fruit directly correlated with the level of *PSY1* expression—highest in *S. lycopersicum*, moderate in *S. cheesmaniae*, and lowest in *S. habrochaites* and *S. pennellii* (Figure 6 and Figure 7; Table 2).

*PSY1* of *S. habrochaites* LA2144 behaved differently from that of *S. habrochaites* LA1777 [33] and similar to the other analyzed GF species in that it was weakly expressed in ripe fruit and produced small amounts of carotenoids (Figure 6 and Figure 7). The latter may be the result of β-carotene biosynthesis in remaining chloroplasts of green fruit tissues due to the expression of both *PSY1* and *PSY2* [12]. Ripe fruit of YF *S. cheesmaniae* had five times lower *PSY1* expression than those of RF *S. lycopersicum* cv. Heinz and accumulated β-carotene instead of lycopene (Figure 6 and Figure 7), which was probably due to increased activity of chromoplast-specific lycopene beta-cyclase caused by polymorphisms [42,48].

The expression level of *PSY1* in RF tomatoes was much higher than in RF *C. annuum* [55], which was thought to compensate for low PSY1 activity in tomato caused by Y136N mutation [10] that was detected in all PSY1 homologs identified in the current study. Two other known substitutions that can affect the level of carotenoid biosynthesis (the C-terminal P to L substitution in grass PSY-E1, found as a result of white endosperm selection, and capable of reducing the biosynthesis of carotenoids in grains [56], and A191D, leading to an increase in PSY enzymatic activity [57]) were not detected in the identified tomato PSY1 homologs.

In wheat, the phenotypic variation of grain yellow pigments is significantly associated with three *Psy1-A1* alleles, differing in the effect on carotenoid content [58]. In the fourth intron of *Psy1-A1a*, related to a large reduction in grain pigment, the 676-bp INDEL was detected, which could be linked to mutations in a regulatory region of the gene that alters its expression [58].

It may be proposed that species-specific differences in *PSY1* expression may depend on variations in the promoter/5′-UTR sequence, in particular, the presence and location of regulatory elements and transcription factor-binding sites. Thus, *S. lycopersicum PSY1* has been shown to contain 5′-UTR with MBS, ABRE, and TC-rich repeats [59]. In the 2.5-kb *PSY1* region comprising the promoter and 5′-UTR, we found 37 types of regulatory elements (Table 3), indicating that *PSY1* transcription may be regulated in response to light, abiotic stresses, and hormones such as methyl jasmonate (MeJA), auxins, abscisic acid (ABA), and ethylene. There were variations in the presence of regulatory elements between RF *S. lycopersicum* and GF *S. pennellii*, such as the loss of some and acquisition of the other light-responsive elements, and the lack of drought-responsive elements in *S. lycopersicum*, which may indicate lower resistance of tomato cultivars to abiotic stresses compared to their wild relatives. ABA is an apo-carotenoid involved in plant developmental processes, including seed maturation and dormancy and stress tolerance [60], whereas JA and auxins together promote resistance to necrotrophic pathogens [61]. The presence of hormone-responsive elements in *PSY1* 5′-UTR/promoter of both RF and GF species indicate the involvement of PSY1 in the regulation of hormone-mediated signaling in tomato. Gibberellin (GAs)-responsive elements have been previously reported only in 5′-UTR of the *S. lycopersicum PSY2* gene [60] but not in the *PSY1* gene, despite the fact that GAs participate in fruit senescence and have the same precursor (GGPP) as carotenoids [61]. Although we also did not identify GAs-responsive elements in the *PSY1* 5′-UTR/promoter, we found them in *PSY1* intron and exon sequences—P-box (CCTTTTG) in introns IV and V of all GF species and GARE (TCTGTTG) in exon I and intron II of both RF and GF species and in intron III of *S. habrochaites* LA2144.

The fruit ripening process, including ethylene production, was shown to be independent of carotenogenesis [62]; nevertheless, we observed that *S. lycopersicum* acquired ethylene-responsive elements in the *PSY1* promoter/5′-UTR (Table 3), which could account for stronger induction of carotenogenesis during fruit ripening compared to *S. pennellii*, and, consequently, contribute to red pigmentation in fruit. 

However, despite these differences, the overall arrangement of regulatory elements in the *PSY1* 5′-UTR/promoter is similar in RF *S. lycopersicum* and GF *S. pennellii*, suggesting a significant degree of conservation of the *PSY1* transcriptional regulatory mechanism between RF and GF species.

## 4. Materials and Methods

### 4.1. Plant Material

Accessions of tomato species (*Solanum* section Lycopersicon) (Table 1) were kindly provided by the Tomato Genetics Resource Center (USA; https://tgrc.ucdavis.edu/). Plants were grown in a greenhouse with temperature kept at 28 °C/23 °C during a 16-h/8-h day/night light cycle (light intensity, 300–400 μmol m^−2^ s^−1^). Young leaves, whole flower buds (closed flowers in the pre-anthesis stage with green petals), colored (yellow) petals, and MG and ripe fruit were collected in two biological replicates and homogenized in liquid nitrogen. The MG stage was defined as firm green fruit of a final (maximal) size, and ripe fruit in GF species was defined by softness and in YF or RF species—by a change of color from green to yellow or red, respectively, due to chlorophyll degradation and carotenoid accumulation [36,63,64].

### 4.2. Gene Identification 

To amplify the full-length *PSY1* genes from tomato species, gene-specific primers (Table 4) were designed based on *PSY1* genomic sequences of *S. lycopersicum* (cv. Heinz 1706) available in NCBI GenBank (Gene ID: 543988, NC_015440.3 [4350836…4355976], http://www.ncbi.nlm.nih.gov/Genbank, genome annotation releases), and Sol Genomics Network (Solyc03g031860.2.1, https://solgenomics.net/); manual revision of sequence polymorphisms and additional evaluation were performed using Primer3 (http://frodo.wi.mit.edu/primer3/). Genomic DNA was isolated from young leaves of a single plant of each species accession, as previously described [65], and used as a template (100 ng) for PCR amplification at the following conditions: initial denaturation at 94 °C for 10 min, 35 cycles of denaturation at 94 °C for 30 s, primer annealing at 55 °C for 30 s, and extension at 65 °C for 4.5 min, and a final extension at 65 °C for 10 min. The amplified PCR products of the expected size were purified by using the QIAEX^®^ II Gel Extraction kit (QIAGEN, Hilden, Germany), cloned in the pGEM^®^-T Easy (Promega, Madison, WI, USA), and sequenced (3–5 clones for each accession) on ABI Prism 377 DNA Sequencer (Applied Biosystems, Waltham, MA, USA) using the designed primers (Table 4).

### 4.3. Structural and Phylogenetic Analysis

Multiple sequence alignments and structural and phylogenetic analyses of *PSY1* genes and encoded proteins were conducted with MEGA 7.0 [66]. Phylogenetic dendrograms were constructed based on complete gene, cDNA, and protein sequences using the neighbor-joining [67] and Jukes-Cantor [68] methods; confidence for tree topologies was estimated by bootstrap values of 1000 replicates. 

Predicted proteins were characterized in terms of molecular weight, isoelectric point (pI) (http://isoelectric.ovh.org; accessed on the 20 March 2020; [69]), secondary, tertiary, and quaternary structures (PHYRE2 [70]; visualization by Chimera-1.11.2, http://www.cgl.ucsf.edu/chimera/download.html; downloaded on February 6, 2017), the functional importance of amino acid residue substitutions (PROVEAN; [71]), and conserved domains, sites, and motifs (NCBI-CDD, https://www.ncbi.nlm.nih.gov/cdd; UniProt, https://www.uniprot.org/; and Multiple Em for Motif Elicitation (MEME 5.1.1) [72], http://meme-suite.org/tools/meme).

For comparative structural analysis, the complete sequences and/or transcripts of *PSY1* gene homologs were extracted from the NCBI database. We used *PSY1* from the model plant *Arabidopsis thaliana* (Gene ID: 831587) and from Solanaceae species: *Solanum* section Petota—*S. tuberosum* (NW_006238968.1: 1057573-1062303), *S. verrucosum* (FYAE01567738.1: 183863-187835) and *S. commersonii* cv. cmm1t (JXZD01122484.1: 1000-7000); other *Solanum* sections—*S. sisymbriifolium* (GGFC02031958.1: 553-1815) *S. melongena* (GBGZ01079994.1: 146-1408), *S. prinophyllum* (374-1636), and *S. torvum* (51-1307); *Capsicum annuum* (NM_001324967.1: 205328571-205334820); *Lycium* species—*L. ruthenicum* (KF957704.1), *L. chinense* (KJ624406.1), and *L. barbarum* (KF957680.1); *Nicotiana tabacum* (GDGU01109046.1: 486-1900); *Petunia axillaris* (GBRU01036418.1: 1-1560).

### 4.4. Gene Expression

Total RNA was isolated from individual samples of young leaves, flower buds with green petals, yellow petals, and fruit using the RNeasy Plant Mini Kit (QIAGEN, Hilden, Germany), qualified by gel electrophoresis, and used for first-strand cDNA synthesis (Reverse Transcription System; Promega, Madison, WI, USA) with an oligo-dT primer. The RNA and cDNA concentrations were quantified by fluorimetry (Qubit^®^ Fluorometer, Thermo Fisher Scientific, Waltham, MA, USA). Quantitative real-time (qRT)-PCR was performed with 2.5 ng of cDNA, SYBR Green RT-PCR mixture (Syntol, Moscow, Russia), and specific primers (Table 4) at the following cycling conditions: initial denaturation at 95 °C for 5 min and 40 cycles of denaturation at 95 °C for 15 s and annealing/extension at 60 °C for 40 s. To normalize the levels of *PSY1* expression, two reference tomato genes *Expressed* (SGN-U346908) and *actin 2/7* (NM_001330119.1) [73,74] were used.

The qRT-PCR results were statistically analyzed with Graph Pad Prism version 7.02 (GraphPad Software Inc., San Diego, CA, USA; https://www.graphpad.com/scientific-software/prism/). The data were expressed as the mean ± standard deviation (SD) based on three technical replicates of two biological replicates. The unequal variance (Welch’s) *t*-test was applied to assess statistical significance (*p*-value < 0.05) of differences in gene expression between tissues within the same species and between the same tissues of different tomato species. 

### 4.5. Carotenoid and Chlorophyll Content

Total carotenoid content was measured by spectrophotometry in two biological and three technical replicates using a modified Folch method [75,76]. Briefly, 0.2 g of plant tissue was homogenized in Folch solution (2:1 chloroform-methanol [v/v]) in the presence of trace Mg_2_CO_3_ amounts [76], incubated at 4 °C for 1 h, and centrifuged at 4000 rpm for 10 min at 4 °C. The lower chloroform phase was collected and used for spectrophotometric assay of chlorophyll, lycopene, β-carotene, and total carotenoid contents were measured in acetone-hexane solutions, as previously described [77] using a spectrophotometer (Basic, Eppendorf, Hamburg, Germany) and the following equations:

Chlorophyll *a* (µg/mL) = 11.47 (A_666_ − A_750_) − 2 (A_648_ − A_750_) Chlorophyll *b* (µg/mL) = 21.85 (A_648_ − A_750_) − 4.53 (A_666_ − A_750_) Total carotenoids (x + c) (µg/mL) = [1000 (A_480_ − A_750_) − 1.33 Chl *a* − 23.93 Chl *b*]/202 Lycopene (mg/100 mL) = 0.204 A_645_ − 0.0458 A_663_ + 0.372 A_505_ − 0.0806 A_453_ β-carotene (mg/100 mL) = 0.216 A_663_ − 1.22 A_645_ − 0.304 A_505_ + 0.452 A_453_

where A_666_, A_648_, A_750_, A_663_, A_645_, A_505_, A_453_ and A_480_ are absorbance values at the indicated wavelengths; x + c—the sum of xanthophylls and carotenes. The extracts were then evaporated in a stream of nitrogen and the pellets were re-dissolved in hexane. The absorption spectrum was measured in the range of 250–800 nm and used for screening for the presence of phytoene and phytofluene based on their distinct spectral signature (maximum absorption of phytoene—at 285 nm, and phytofluene—at 331, 347 and 365 nm) in the UV range (Supplementary file) [78].

### 4.6. Promoter and 5′-UTR Analyses

1. The search of specific cis-elements in promoters and 5′-UTRs was performed using the PlantCARE database, which provides an evaluation of cis-regulatory elements, enhancers, and repressors [79]; (http://bioinformatics.psb.ugent.be/webtools/plantcare/html/; accessed May 31, 2020).

## 5. Conclusions

In this study, we identified *PSY1* homologous genes in RF, YF, and GF tomato species of *Solanum* section Lycopersicon. *PSY1* homologs shared high similarity of structure, conserved motifs, and functionally important sites. *PSY1* transcription levels were species- and plant organ-specific, and directly correlated with carotenoid content in ripe fruit. Analysis of the *PSY1* promoter and 5′-UTR sequences revealed differences between GF and RF tomatoes, which could be attributed to domestication. Our results provide valuable data for further functional and evolutionary characterization of the carotenogenesis pathway in fleshy fruit in the Solanaceae family.

## Figures and Tables

**Table 1:** Characteristics of *PSY1* homologs from the analyzed *Solanum* section Lycopersicon species.

Accession	TGRC Collection Number	Origin	Ripe Fruit Color *	NCBI Gene ID/Solyc No.	Gene, bp	cDNA, bp	Protein, aa	pI	MW, kDa
*PSY1* genes identified in this study									
*S. lycopersicum* L. cv. Heinz 1706-BG (*Lycopersicon* group)	LA4345		Red	MT664042	4871	1239	412	8.1	46.6
*S. cheesmaniae* (L. Riley) Fosberg (Esculenthum group/*Lycopersicon* group)	LA0421	San Cristobal: cliff East of Wreck Bay, Galapagos Islands, Ecuador	Yellow	MN782521 MN782522	4883/4876	1239	412	7.74/7.9	46.6/45.5
*S. chilense* (Dunal) Reiche (Peruvianum group/*Eriopersicon* group)	LA1963	Rio Caplina, Tacna, Peru	Green to whitish green with purple stripes	MN782523 MN812838	4858/4840	1239	412	7.74/7.29	46.6/46.3
	LA2884	Ayaviri, Antofagasta, Chile		MN782524 MN782525	4878/4876	1239	412	7.93/7.90	46.5/46.5
*S. habrochaites* S. Knapp & D. M. Spooner (Hirsutum group/*Eriopersicon* group)	LA1771	Rio Casma, Ancash, Peru	Green with darker green stripes	MN782526 MN782527	4914/4916	1239	412	8.24	46.6
	LA2144	Chanchan, Chimborazo, Ecuador		MN782528 MN782529	4906/4903	1239	412	8.1	46.6
*S. pennellii* Correll (Hirsutum group/*Neolycopersicon* group)	LA1926	Agua Pertida, Ica, Peru	Green	MN782530 MN782531	4898/4901	1239	412	8.5/8.1	46.7/46.6
	LA0716	Atico, Arequipa, Peru		MN782532	4886	1239	412	8.1	46.5
*PSY1* genes available in NCBI GenBank									
*S. lycopersicum* cv. Heinz 1706			Red	543988, NC_015440.3 (4350836..4355976); Solyc03g031860.2.1	4872	1239	412	8.1	46.6
*S. pimpinellifolium*	LA1589	La Libertad, Peru	Red	AGFK01024844.1 (1289..7095)	4872	1239	412	8.1	46.6
*S. arcanum*	LA2157	Tunel Chotano, Cajamarica, Peru	Green	CBYQ010012533.1 (26020..31886)	4880	1239	412	8.1	46.6
*S. habrochaites*	LYC4	Peru/Ecuador	Green with dark-green stripes	CBYS010011028.1 (46212..52055)	4910	1239	412	8.1	46.6
*S. pennellii*	LA0716	Atico, Arequipa, Peru	Green	CCXL01009615.1 (3669..9559)	4949	1239	412	8.1	46.6

* Fruit color data are indicated according to ref. [36]. pI—isoelectric point; MW—molecular weight; gene sequences were found in the NCBI database.

**Table 2:** Chlorophyll and carotenoid content in ripe fruits and leaves of analyzed tomato accessions.

Accession	Ripe Fruit Pigment Content, µg/g FW					Leaf Pigment Content, µg/g FW	
	Chlorophyll (a + b)	Lycopene	Total Carotenoids (x + c)	β-carotene	Other x + c (-β-carotene)	Total Carotenoids (x + c)	Chlorophyll (a + b)
*Solanum lycopersicum* cv. Heinz 1706	N/D	0.09 ± 0.02	0.24 ± 0.030	0.01 ± 0.008	~0.23	0.94 ± 0.098	3.19 ± 0.390
*Solanum cheesmaniae* LA 0421	N/D	N/D	0.03 ± 0.004	0.02 ± 0.003	~0.01	0.96 ± 0.210	3.33 ± 0.820
*Solanum chilense* LA 1963	0.03 ± 0.004	N/D	0.01 ± 0.004	0.01 ± 0.001	~0.00	0.89 ± 0.070	2.78 ± 0.260
*Solanum habrochaites* LA 2144	0.06 ± 0.010	N/D	0.02 ± 0.006	0.01 ± 0.003	~0.00	0.93 ± 0.004	3.15 ± 0.060
*Solanum pennellii* LA 0716	0.09 ± 0.050	N/D	0.02 ± 0.005	0.02 ± 0.004	~0.00	0.96 ± 0.110	3.51 ± 0.400

N/D—not detected; FW—fresh weight.

**Table 3:** Regulatory elements found in the *PSY1* promoter and 5′-UTR of RF *S. lycopersicum* and GF *S. pennellii*.

No	Type	Sequence	*S. lycopersicum* cv. Heinz 1706		*S. pennellii* LA0716		Comments
			Strand	Position	Strand	Position	
1	chs-Unit 1 m1		**n/d**		**-**	**−1110**	Part of a light responsive element
2	Box II	ACACGTAGA	-	−1713	-	−1735	
3	GATA-motif	AAGGATAAGG	**+**	**−2207**	**n/d**		
4	GTGGC-motif	GATTCTGTGGC	+	−564	+	−566	
5	TCT-motif		**n/d**		**+**	**−2419**	
6	AE-box	AGAAACAA	-	−528	+	−530	Part of a conserved DNA module involved in light response
7	Box 4	ATTAAT	+	−2373	+	−2402	
			+	−1685	+	−1707	
8	GA-motif	ATAGATAA	**n/d**		**-**	**−2143**	
9	I-box	TAGATAACC	**n/d**		**+**	**−28**	
10	3-AF3 binding site	CACTATCTAAC	**+**	**−2321**	**n/d**		Light response
11	GT1-motif	GGTTAA	+	−1101	+	−1102	
			**n/d**		**+**	**−1079**	
12	ABRE	TACGTGTC	+	−1711	+	−1733	The binding sites for AREB/ABF factors involved in the abscisic acid response
		ACGTG	+	−675	+	−677	
			+	−1710	+	−1732	
13	ABRE3a	TACGTG	+	−1711	+	−1733	
			+	−676	+	−678	
14	ABRE4	CACGTA	-	−1711	-	−1733	
			-	−676	-	−678	
15	AT~ABRE	TACGTGTC	+	−1711	+	−1733	
16	ERE	ATTTTAAA	**+**	**−1272**	**n/d**		Ethylene-response
17	TGA-element	AACGAC	+	−2241	+	−2270	Auxin-response
			+	−2219	+	−2248	
18	CGTCA-motif	CGTCA	-	−1846	-	−1876	MeJA response
19	STRE	AGGGG	**+**	**−1384**	**n/d**		Defense and stress response
			**-**	**−1459**			
			+	−712	+	−714	
20	TC-rich repeats	GTTTTCTTAC	+	−16	+	−16	
21	ARE	AAACCA	**-**	**−1988**	**n/d**		Essential for the anaerobic induction
			**+**	**−1964**			
			-	−265	-	−265	
			+	−1527	+	−1560	
			-	−204	-	−204	
			-	−682	-	−684	
22	WUN-motif	AAATTACT	-	−102	-	−102	Wounding response
23	CAT-box	GCCACT	-	−1496	-	−1495	Related to meristem-specific expression
			**-**	**−1221**	**n/d**		
			**n/d**		**-**	**−1528**	
24	AT~TATA-box	TATATA	**+**	**−1391**	**n/d**		Enriched near transcription start. TATA-box-like, putative TBP-binding
			**-**	**−973**			
			-	−1007	-	−1007	
			-	−1159	-	−1164	
25	CAAT-box	CAAT/CAAAT	43 repeats		42 repeats		Common cis-acting element in promoter and enhancer
26	TATA-box	TATAAAAT; TATAAATA; TATAAAT; TATAAA; TATAA; TATA	multiple repeats		multiple repeats		Core promoter element
27	W box	TTGACC	-	−1894	-	−1924	WRKY TF binding site
28	MBS	CAACTG	**n/d**		**-**	**−1825**	MYB TF binding site; drought response
29	MRE	AACCTAA	-	−2275	-	−2286	MYB TF binding site; light response
			**n/d**		**-**	**−580**	
					**+**	**−2304**	
30	MYB	TAACTG	+	−551	+	−553	MYB TF binding site
			**n/d**		**-**	**−1825**	
			**+**	**−289**	**n/d**		
		CAACAG	-	−517	-	−519	
			**n/d**		**-**	**−1538**	
		CAACCA	**+**	**−24**	**n/d**		
		TAACCA	-	−309	-	−311	
			**n/d**		**+**	**−24**	
31	MYC	CATGTG	-	−787	-	−788	
			-	−278	-	−278	
32	AS-1 (activation sequence-1)	TGACG	+	−1846	+	−1876	Originally found in some viral and bacterial T-DNA promoters. Pathogen-inducible
33	AT1-motif	AATTATTTTTTATT	**-**	**−2111**	**n/d**		Binding site of AT-rich DNA binding protein (ATBP-1)
34	G-box	TACGTG	+	−1711	+	−1733	Multifunctional
			+	−676	+	−678	
35	Unnamed__2	AACCTAACCT	**-**	**−1107**	**n/d**		Unknown function
36	Unnamed__4	CTCC	13 repeats		13 repeats		
37	AAGAA-motif	GAAAGAA	**n/d**		**+**	**−1515**	

The elements that are different (present or not) between the two species are highlighted in bold and pink.

**Table 4:** List of primers for *PSY1* gene amplification, sequencing, and expression analysis.

Primer	Sequence (5′→3’)	Application
PSY1geneF	AGTGGGAATCTACTAGGAGT	Gene amplification and sequencing
PSY1geneR	TTATCTTTGAAGAGAGACAGTTT	
tPSY_F6	CTATCTGGGCAATATATGGTG	Gene sequencing
tPSY_F7	TCTCGTCCTAGATACTACAC	
tPSY_F8	CAGTGACGAGCCATGATC	
tPSY_F9	TTGAGCTTGTCGTTCTCAGT	
PSY1rtF	CTGAGATCTACCAATGAGTTAG	qRT-PCR, *PSY1*
PSY1rtR	TCTCGGGAGTCATTAGCATAG	
prPSY1-F	GTTGGATTTGCATGTAGACC	Promoter/5′-UTR amplification and sequencing
tPSY_F2	GGCTAAATCGAAAATYGAATC	
tPSY_F3	TAACTTTCTATTGCTTTGCTAGTG	
tPSY_F4	TGGTAGGTAATATTGCTGATTTTG	
Actin 2/7-F	CATTGTGCTCAGTGGTGGTTC	qRT-PCR, reference genes
Actin 2/7F-R	TCTGCTGGAAGGTGCTAAGTG	
Expressed-F	GCTAAGAACGCTGGACCTAATG	
Expressed-R	TGGGTGTGCCTTTCTGAATG	

## Supplementary Materials

The following are available online at https://www.mdpi.com/2223-7747/9/9/1169/s1.
