# Supplementary material for: Differential Regulation of Phytoene Synthase PSY1 During Fruit Carotenogenesis in Cultivated and Wild Tomato Species (Solanum section Lycopersicon)
Source: Plants (Basel). 2020 Sep 9;9(9):1169. doi: 10.3390/plants9091169 (PMC7569967; doi:10.3390/plants9091169)
Supplement: Supplementary file 1 [file plants-09-01169-s001.pdf]

Eppendorf BioSpectrometer basic

Device # : 6135HK604082

Software # : 4.3.6.0

2020/8/25 ***250_800 2020-08-25 15:05:19***

				**GF**	**GF**
Sample ID:		Hexane	Hexane	*S. habrochaites* LA 2144	*S. pennellii* LA0716
Sample No:		001	002	003	004
	Dilution			no	no
	Fresh weight, mg			213,9	257,2
	Hexane volume, ml			1	1
Wavelength (nm)					
250	0.000	-0.005	-0.003	0.747	0.990
251	0.000	-0.005	-0.003	0.734	0.980
252	0.000	-0.005	-0.002	0.723	0.970
253	0.000	-0.005	-0.002	0.711	0.962
254	0.000	-0.005	-0.002	0.700	0.955
255	0.000	-0.005	-0.002	0.691	0.949
256	0.000	-0.006	-0.002	0.682	0.944
257	0.000	-0.005	-0.002	0.675	0.939
258	0.000	-0.004	-0.001	0.668	0.936
259	0.000	-0.003	-0.001	0.661	0.932
260	0.000	-0.004	-0.002	0.654	0.929
261	0.000	-0.005	-0.002	0.647	0.926
262	0.000	-0.005	-0.003	0.642	0.923
263	0.000	-0.006	-0.003	0.636	0.920
264	0.000	-0.006	-0.003	0.632	0.918
265	0.000	-0.006	-0.002	0.627	0.915
266	0.000	-0.006	-0.002	0.621	0.912
267	0.000	-0.006	-0.002	0.614	0.905
268	0.000	-0.006	-0.002	0.607	0.897
269	0.000	-0.006	-0.002	0.599	0.887
270	0.000	-0.007	-0.003	0.593	0.875
271	0.000	-0.007	-0.003	0.587	0.865
272	0.000	-0.007	-0.003	0.581	0.854
273	0.000	-0.008	-0.003	0.577	0.844
274	0.000	-0.008	-0.003	0.571	0.833
275	0.000	-0.009	-0.003	0.564	0.821
276	0.000	-0.009	-0.003	0.554	0.806
277	0.000	-0.008	-0.003	0.542	0.790
278	0.000	-0.009	-0.003	0.530	0.776
279	0.000	-0.009	-0.003	0.519	0.763
280	0.000	-0.010	-0.003	0.509	0.752
281	0.000	-0.009	-0.003	0.501	0.742
282	0.000	-0.009	-0.003	0.493	0.733
283	0.000	-0.009	-0.003	0.484	0.722
284	0.000	-0.009	-0.004	0.474	0.710

285	0.000	-0.009	-0.004	0.462	0.696
286	0.000	-0.009	-0.004	0.450	0.682
287	0.000	-0.009	-0.003	0.437	0.668
288	0.000	-0.008	-0.003	0.426	0.658
289	0.000	-0.008	-0.003	0.415	0.648
290	0.000	-0.008	-0.003	0.405	0.639
291	0.000	-0.008	-0.003	0.395	0.629
292	0.000	-0.008	-0.003	0.386	0.618
293	0.000	-0.008	-0.003	0.378	0.605
294	0.000	-0.008	-0.003	0.370	0.592
295	0.000	-0.009	-0.003	0.363	0.580
296	0.000	-0.009	-0.003	0.357	0.569
297	0.000	-0.009	-0.003	0.351	0.560
298	0.000	-0.009	-0.003	0.345	0.552
299	0.000	-0.009	-0.003	0.340	0.545
300	0.000	-0.009	-0.003	0.336	0.539
301	0.000	-0.009	-0.003	0.332	0.535
302	0.000	-0.009	-0.003	0.328	0.530
303	0.000	-0.009	-0.003	0.324	0.526
304	0.000	-0.008	-0.003	0.320	0.521
305	0.000	-0.008	-0.003	0.316	0.517
306	0.000	-0.008	-0.003	0.312	0.512
307	0.000	-0.007	-0.003	0.308	0.507
308	0.000	-0.007	-0.002	0.304	0.503
309	0.000	-0.007	-0.002	0.301	0.499
310	0.000	-0.007	-0.003	0.298	0.496
311	0.000	-0.007	-0.003	0.296	0.494
312	0.000	-0.007	-0.003	0.294	0.492
313	0.000	-0.007	-0.003	0.293	0.491
314	0.000	-0.007	-0.003	0.291	0.490
315	0.000	-0.007	-0.003	0.289	0.490
316	0.000	-0.007	-0.003	0.287	0.489
317	0.000	-0.007	-0.003	0.285	0.488
318	0.000	-0.007	-0.003	0.283	0.488
319	0.000	-0.008	-0.003	0.281	0.488
320	0.000	-0.008	-0.003	0.280	0.488
321	0.000	-0.007	-0.002	0.278	0.488
322	0.000	-0.007	-0.002	0.277	0.488
323	0.000	-0.007	-0.003	0.275	0.489
324	0.000	-0.007	-0.003	0.274	0.491
325	0.000	-0.007	-0.002	0.273	0.493
326	0.000	-0.007	-0.002	0.272	0.495
327	0.000	-0.008	-0.003	0.271	0.497
328	0.000	-0.008	-0.003	0.270	0.499
329	0.000	-0.008	-0.003	0.269	0.501
330	0.000	-0.007	-0.003	0.268	0.502
331	0.000	-0.006	-0.002	0.266	0.502
332	0.000	-0.006	-0.002	0.264	0.502
333	0.000	-0.006	-0.002	0.262	0.501
334	0.000	-0.006	-0.002	0.260	0.498
335	0.000	-0.007	-0.003	0.258	0.496
336	0.000	-0.007	-0.003	0.256	0.493
337	0.000	-0.007	-0.003	0.254	0.489
338	0.000	-0.006	-0.003	0.251	0.485
339	0.000	-0.006	-0.003	0.250	0.482
340	0.000	-0.005	-0.002	0.249	0.479

341	0.000	-0.005	-0.002	0.247	0.475
342	0.000	-0.006	-0.002	0.245	0.470
343	0.000	-0.006	-0.002	0.243	0.466
344	0.000	-0.005	-0.002	0.242	0.462
345	0.000	-0.005	-0.002	0.241	0.458
346	0.000	-0.005	-0.002	0.240	0.454
347	0.000	-0.006	-0.002	0.239	0.450
348	0.000	-0.007	-0.003	0.239	0.448
349	0.000	-0.006	-0.003	0.239	0.446
350	0.000	-0.006	-0.002	0.239	0.445
351	0.000	-0.005	-0.002	0.240	0.445
352	0.000	-0.005	-0.002	0.243	0.446
353	0.000	-0.005	-0.002	0.245	0.448
354	0.000	-0.006	-0.002	0.247	0.451
355	0.000	-0.007	-0.002	0.249	0.454
356	0.000	-0.007	-0.003	0.251	0.458
357	0.000	-0.008	-0.003	0.254	0.462
358	0.000	-0.008	-0.003	0.256	0.467
359	0.000	-0.008	-0.003	0.260	0.472
360	0.000	-0.008	-0.003	0.264	0.478
361	0.000	-0.009	-0.003	0.268	0.484
362	0.000	-0.009	-0.003	0.271	0.489
363	0.000	-0.009	-0.004	0.274	0.495
364	0.000	-0.009	-0.004	0.277	0.500
365	0.000	-0.009	-0.004	0.282	0.506
366	0.000	-0.008	-0.003	0.286	0.513
367	0.000	-0.006	-0.003	0.291	0.520
368	0.000	-0.006	-0.003	0.296	0.527
369	0.000	-0.006	-0.003	0.301	0.536
370	0.000	-0.005	-0.002	0.305	0.545
371	0.000	-0.006	-0.002	0.310	0.553
372	0.000	-0.005	-0.002	0.314	0.562
373	0.000	-0.004	-0.002	0.319	0.571
374	0.000	-0.004	-0.002	0.322	0.579
375	0.000	-0.006	-0.002	0.325	0.586
376	0.000	-0.005	-0.002	0.328	0.593
377	0.000	-0.005	-0.002	0.331	0.600
378	0.000	-0.006	-0.002	0.333	0.607
379	0.000	-0.006	-0.002	0.336	0.613
380	0.000	-0.006	-0.002	0.338	0.618
381	0.000	-0.006	-0.003	0.340	0.624
382	0.000	-0.005	-0.002	0.342	0.629
383	0.000	-0.004	-0.002	0.344	0.634
384	0.000	-0.004	-0.002	0.347	0.638
385	0.000	-0.004	-0.002	0.349	0.641
386	0.000	-0.004	-0.002	0.351	0.644
387	0.000	-0.005	-0.002	0.353	0.647
388	0.000	-0.004	-0.002	0.356	0.650
389	0.000	-0.004	-0.002	0.359	0.652
390	0.000	-0.004	-0.002	0.362	0.655
391	0.000	-0.005	-0.002	0.365	0.658
392	0.000	-0.005	-0.002	0.369	0.661
393	0.000	-0.005	-0.002	0.374	0.665
394	0.000	-0.005	-0.002	0.379	0.670
395	0.000	-0.005	-0.002	0.386	0.676
396	0.000	-0.005	-0.003	0.393	0.685

397	0.000	-0.004	-0.002	0.400	0.695
398	0.000	-0.004	-0.002	0.409	0.708
399	0.000	-0.003	-0.002	0.419	0.723
400	0.000	-0.003	-0.002	0.430	0.742
401	0.000	-0.003	-0.002	0.441	0.762
402	0.000	-0.003	-0.002	0.453	0.785
403	0.000	-0.003	-0.002	0.465	0.808
404	0.000	-0.003	-0.002	0.476	0.833
405	0.000	-0.003	-0.002	0.487	0.858
406	0.000	-0.003	-0.002	0.498	0.882
407	0.000	-0.003	-0.002	0.509	0.906
408	0.000	-0.003	-0.002	0.519	0.930
409	0.000	-0.003	-0.002	0.528	0.952
410	0.000	-0.003	-0.002	0.537	0.973
411	0.000	-0.003	-0.002	0.544	0.992
412	0.000	-0.003	-0.002	0.550	1.008
413	0.000	-0.003	-0.002	0.554	1.022
414	0.000	-0.003	-0.002	0.557	1.034
415	0.000	-0.003	-0.002	0.560	1.045
416	0.000	-0.003	-0.002	0.562	1.053
417	0.000	-0.003	-0.002	0.565	1.061
418	0.000	-0.002	-0.002	0.569	1.068
419	0.000	-0.002	-0.002	0.573	1.077
420	0.000	-0.003	-0.002	0.579	1.090
421	0.000	-0.003	-0.002	0.588	1.105
422	0.000	-0.003	-0.002	0.597	1.123
423	0.000	-0.002	-0.002	0.608	1.144
424	0.000	-0.003	-0.002	0.619	1.168
425	0.000	-0.003	-0.003	0.631	1.198
426	0.000	-0.003	-0.002	0.643	1.229
427	0.000	-0.003	-0.002	0.654	1.263
428	0.000	-0.003	-0.002	0.665	1.297
429	0.000	-0.003	-0.002	0.673	1.331
430	0.000	-0.003	-0.002	0.679	1.364
431	0.000	-0.003	-0.002	0.681	1.393
432	0.000	-0.003	-0.002	0.678	1.416
433	0.000	-0.003	-0.002	0.671	1.432
434	0.000	-0.002	-0.002	0.659	1.437
435	0.000	-0.002	-0.002	0.643	1.433
436	0.000	-0.002	-0.002	0.624	1.419
437	0.000	-0.002	-0.002	0.603	1.393
438	0.000	-0.002	-0.002	0.581	1.360
439	0.000	-0.002	-0.002	0.560	1.320
440	0.000	-0.002	-0.002	0.540	1.276
441	0.000	-0.002	-0.002	0.522	1.229
442	0.000	-0.003	-0.002	0.506	1.180
443	0.000	-0.003	-0.002	0.492	1.132
444	0.000	-0.003	-0.002	0.480	1.086
445	0.000	-0.003	-0.002	0.469	1.039
446	0.000	-0.002	-0.002	0.459	0.996
447	0.000	-0.002	-0.002	0.451	0.960
448	0.000	-0.002	-0.002	0.445	0.929
449	0.000	-0.002	-0.002	0.439	0.902
450	0.000	-0.002	-0.002	0.434	0.879
451	0.000	-0.002	-0.002	0.429	0.861
452	0.000	-0.002	-0.002	0.424	0.846

453	0.000	-0.002	-0.002	0.420	0.836
454	0.000	-0.002	-0.002	0.416	0.829
455	0.000	-0.002	-0.002	0.413	0.825
456	0.000	-0.002	-0.002	0.409	0.825
457	0.000	-0.002	-0.002	0.407	0.826
458	0.000	-0.002	-0.002	0.404	0.830
459	0.000	-0.002	-0.002	0.402	0.833
460	0.000	-0.002	-0.002	0.400	0.838
461	0.000	-0.002	-0.002	0.398	0.843
462	0.000	-0.002	-0.002	0.397	0.849
463	0.000	-0.003	-0.002	0.394	0.853
464	0.000	-0.003	-0.002	0.392	0.855
465	0.000	-0.003	-0.002	0.388	0.854
466	0.000	-0.003	-0.002	0.383	0.851
467	0.000	-0.003	-0.002	0.378	0.845
468	0.000	-0.003	-0.002	0.372	0.834
469	0.000	-0.003	-0.002	0.364	0.819
470	0.000	-0.003	-0.003	0.356	0.801
471	0.000	-0.003	-0.002	0.346	0.779
472	0.000	-0.002	-0.002	0.335	0.754
473	0.000	-0.002	-0.002	0.322	0.724
474	0.000	-0.002	-0.002	0.309	0.692
475	0.000	-0.002	-0.002	0.295	0.658
476	0.000	-0.002	-0.002	0.280	0.622
477	0.000	-0.002	-0.002	0.265	0.586
478	0.000	-0.002	-0.002	0.250	0.550
479	0.000	-0.002	-0.002	0.235	0.513
480	0.000	-0.002	-0.002	0.220	0.476
481	0.000	-0.002	-0.002	0.204	0.439
482	0.000	-0.002	-0.002	0.188	0.403
483	0.000	-0.002	-0.002	0.174	0.370
484	0.000	-0.002	-0.002	0.161	0.342
485	0.000	-0.002	-0.002	0.150	0.317
486	0.000	-0.002	-0.002	0.139	0.293
487	0.000	-0.003	-0.003	0.129	0.270
488	0.000	-0.002	-0.003	0.119	0.248
489	0.000	-0.002	-0.002	0.110	0.228
490	0.000	-0.002	-0.003	0.101	0.209
491	0.000	-0.002	-0.003	0.094	0.191
492	0.000	-0.002	-0.002	0.087	0.176
493	0.000	-0.002	-0.002	0.081	0.162
494	0.000	-0.002	-0.002	0.075	0.150
495	0.000	-0.002	-0.002	0.070	0.138
496	0.000	-0.002	-0.002	0.065	0.128
497	0.000	-0.002	-0.002	0.061	0.118
498	0.000	-0.002	-0.002	0.057	0.110
499	0.000	-0.002	-0.002	0.053	0.102
500	0.000	-0.002	-0.002	0.050	0.096
501	0.000	-0.002	-0.002	0.047	0.090
502	0.000	-0.002	-0.001	0.045	0.085
503	0.000	-0.002	-0.002	0.043	0.080
504	0.000	-0.002	-0.002	0.041	0.075
505	0.000	-0.002	-0.002	0.040	0.071
506	0.000	-0.002	-0.002	0.037	0.067
507	0.000	-0.002	-0.002	0.036	0.063
508	0.000	-0.002	-0.002	0.035	0.061

509	0.000	-0.002	-0.002	0.034	0.058
510	0.000	-0.002	-0.002	0.032	0.055
511	0.000	-0.002	-0.002	0.031	0.053
512	0.000	-0.002	-0.002	0.030	0.051
513	0.000	-0.002	-0.002	0.028	0.050
514	0.000	-0.002	-0.002	0.027	0.049
515	0.000	-0.002	-0.002	0.027	0.047
516	0.000	-0.002	-0.002	0.027	0.046
517	0.000	-0.002	-0.002	0.026	0.045
518	0.000	-0.002	-0.002	0.026	0.045
519	0.000	-0.002	-0.002	0.025	0.044
520	0.000	-0.002	-0.002	0.026	0.044
521	0.000	-0.003	-0.002	0.026	0.044
522	0.000	-0.003	-0.002	0.026	0.045
523	0.000	-0.002	-0.001	0.027	0.046
524	0.000	-0.002	-0.002	0.028	0.046
525	0.000	-0.002	-0.002	0.029	0.047
526	0.000	-0.002	-0.002	0.029	0.048
527	0.000	-0.002	-0.002	0.029	0.048
528	0.000	-0.002	-0.003	0.029	0.048
529	0.000	-0.002	-0.002	0.030	0.049
530	0.000	-0.002	-0.003	0.031	0.049
531	0.000	-0.003	-0.003	0.031	0.049
532	0.000	-0.003	-0.003	0.033	0.050
533	0.000	-0.002	-0.003	0.034	0.051
534	0.000	-0.002	-0.003	0.034	0.051
535	0.000	-0.002	-0.003	0.034	0.052
536	0.000	-0.003	-0.003	0.034	0.053
537	0.000	-0.002	-0.002	0.033	0.053
538	0.000	-0.002	-0.002	0.032	0.053
539	0.000	-0.002	-0.002	0.032	0.053
540	0.000	-0.002	-0.002	0.031	0.053
541	0.000	-0.002	-0.002	0.031	0.053
542	0.000	-0.002	-0.002	0.030	0.053
543	0.000	-0.003	-0.003	0.029	0.052
544	0.000	-0.003	-0.003	0.028	0.052
545	0.000	-0.003	-0.003	0.028	0.051
546	0.000	-0.003	-0.003	0.027	0.052
547	0.000	-0.003	-0.002	0.027	0.052
548	0.000	-0.002	-0.002	0.027	0.052
549	0.000	-0.003	-0.002	0.027	0.051
550	0.000	-0.003	-0.002	0.026	0.050
551	0.000	-0.006	-0.002	0.024	0.049
552	0.000	-0.007	-0.002	0.024	0.049
553	0.000	-0.006	-0.002	0.024	0.050
554	0.000	-0.006	-0.002	0.025	0.050
555	0.000	-0.006	-0.002	0.025	0.051
556	0.000	-0.007	-0.002	0.025	0.052
557	0.000	-0.007	-0.002	0.026	0.053
558	0.000	-0.007	-0.003	0.026	0.054
559	0.000	-0.007	-0.003	0.027	0.055
560	0.000	-0.007	-0.003	0.028	0.056
561	0.000	-0.007	-0.003	0.029	0.058
562	0.000	-0.006	-0.002	0.031	0.060
563	0.000	-0.006	-0.002	0.032	0.063
564	0.000	-0.006	-0.002	0.033	0.065

565	0.000	-0.007	-0.002	0.034	0.067
566	0.000	-0.007	-0.002	0.034	0.069
567	0.000	-0.008	-0.003	0.035	0.071
568	0.000	-0.008	-0.003	0.036	0.074
569	0.000	-0.007	-0.002	0.038	0.076
570	0.000	-0.006	-0.002	0.038	0.078
571	0.000	-0.007	-0.002	0.039	0.080
572	0.000	-0.007	-0.002	0.040	0.083
573	0.000	-0.007	-0.002	0.041	0.085
574	0.000	-0.007	-0.002	0.042	0.087
575	0.000	-0.007	-0.002	0.042	0.089
576	0.000	-0.007	-0.002	0.042	0.091
577	0.000	-0.007	-0.003	0.042	0.093
578	0.000	-0.007	-0.003	0.043	0.095
579	0.000	-0.007	-0.003	0.043	0.096
580	0.000	-0.007	-0.003	0.043	0.097
581	0.000	-0.008	-0.003	0.043	0.098
582	0.000	-0.008	-0.003	0.043	0.099
583	0.000	-0.007	-0.002	0.043	0.100
584	0.000	-0.006	-0.002	0.043	0.100
585	0.000	-0.006	-0.002	0.043	0.100
586	0.000	-0.006	-0.002	0.044	0.101
587	0.000	-0.006	-0.002	0.043	0.101
588	0.000	-0.007	-0.003	0.043	0.101
589	0.000	-0.007	-0.003	0.044	0.102
590	0.000	-0.005	-0.002	0.044	0.103
591	0.000	-0.005	-0.002	0.046	0.103
592	0.000	-0.005	-0.002	0.046	0.104
593	0.000	-0.005	-0.002	0.047	0.106
594	0.000	-0.006	-0.002	0.048	0.106
595	0.000	-0.006	-0.002	0.049	0.107
596	0.000	-0.007	-0.002	0.049	0.109
597	0.000	-0.007	-0.002	0.051	0.111
598	0.000	-0.007	-0.002	0.052	0.113
599	0.000	-0.007	-0.003	0.054	0.115
600	0.000	-0.008	-0.003	0.055	0.117
601	0.000	-0.008	-0.002	0.057	0.121
602	0.000	-0.007	-0.002	0.060	0.125
603	0.000	-0.007	-0.002	0.062	0.129
604	0.000	-0.007	-0.002	0.064	0.132
605	0.000	-0.007	-0.003	0.066	0.135
606	0.000	-0.007	-0.003	0.067	0.138
607	0.000	-0.007	-0.004	0.069	0.141
608	0.000	-0.007	-0.003	0.071	0.144
609	0.000	-0.006	-0.003	0.072	0.148
610	0.000	-0.007	-0.003	0.073	0.151
611	0.000	-0.006	-0.003	0.075	0.155
612	0.000	-0.006	-0.002	0.076	0.159
613	0.000	-0.007	-0.003	0.076	0.161
614	0.000	-0.007	-0.003	0.076	0.162
615	0.000	-0.008	-0.003	0.075	0.164
616	0.000	-0.008	-0.003	0.075	0.165
617	0.000	-0.007	-0.003	0.077	0.167
618	0.000	-0.007	-0.003	0.078	0.169
619	0.000	-0.007	-0.002	0.077	0.169
620	0.000	-0.006	-0.002	0.076	0.169

621	0.000	-0.006	-0.002	0.074	0.168
622	0.000	-0.006	-0.002	0.073	0.168
623	0.000	-0.006	-0.002	0.073	0.167
624	0.000	-0.006	-0.002	0.071	0.166
625	0.000	-0.006	-0.002	0.071	0.164
626	0.000	-0.007	-0.002	0.070	0.162
627	0.000	-0.008	-0.003	0.069	0.160
628	0.000	-0.007	-0.003	0.068	0.160
629	0.000	-0.006	-0.002	0.068	0.160
630	0.000	-0.005	-0.002	0.070	0.160
631	0.000	-0.006	-0.003	0.072	0.159
632	0.000	-0.006	-0.003	0.073	0.160
633	0.000	-0.007	-0.003	0.074	0.162
634	0.000	-0.007	-0.003	0.077	0.165
635	0.000	-0.008	-0.003	0.081	0.169
636	0.000	-0.007	-0.003	0.085	0.173
637	0.000	-0.007	-0.003	0.090	0.181
638	0.000	-0.007	-0.002	0.097	0.191
639	0.000	-0.007	-0.002	0.104	0.201
640	0.000	-0.006	-0.002	0.111	0.213
641	0.000	-0.006	-0.002	0.118	0.225
642	0.000	-0.006	-0.002	0.124	0.237
643	0.000	-0.007	-0.002	0.131	0.251
644	0.000	-0.007	-0.002	0.138	0.268
645	0.000	-0.007	-0.002	0.146	0.282
646	0.000	-0.008	-0.002	0.152	0.297
647	0.000	-0.008	-0.002	0.158	0.312
648	0.000	-0.007	-0.002	0.166	0.330
649	0.000	-0.007	-0.004	0.176	0.349
650	0.000	-0.007	-0.004	0.185	0.369
651	0.000	-0.006	-0.003	0.198	0.392
652	0.000	-0.006	-0.003	0.212	0.416
653	0.000	-0.006	-0.002	0.229	0.444
654	0.000	-0.007	-0.003	0.246	0.475
655	0.000	-0.007	-0.002	0.267	0.509
656	0.000	-0.006	-0.002	0.290	0.545
657	0.000	-0.007	-0.002	0.312	0.579
658	0.000	-0.006	-0.002	0.332	0.614
659	0.000	-0.007	-0.002	0.351	0.646
660	0.000	-0.007	-0.003	0.365	0.673
661	0.000	-0.009	-0.004	0.373	0.692
662	0.000	-0.010	-0.005	0.375	0.706
663	0.000	-0.009	-0.006	0.373	0.714
664	0.000	-0.008	-0.005	0.367	0.714
665	0.000	-0.008	-0.003	0.356	0.708
666	0.000	-0.007	-0.002	0.342	0.694
667	0.000	-0.008	-0.002	0.323	0.671
668	0.000	-0.008	-0.002	0.302	0.642
669	0.000	-0.008	-0.003	0.280	0.610
670	0.000	-0.007	-0.003	0.258	0.576
671	0.000	-0.008	-0.003	0.233	0.537
672	0.000	-0.007	-0.002	0.210	0.496
673	0.000	-0.007	-0.002	0.185	0.455
674	0.000	-0.007	-0.002	0.163	0.413
675	0.000	-0.007	-0.002	0.145	0.372
676	0.000	-0.007	-0.003	0.127	0.331

677	0.000	-0.006	-0.003	0.109	0.293
678	0.000	-0.006	-0.002	0.093	0.258
679	0.000	-0.007	-0.001	0.080	0.225
680	0.000	-0.007	-0.002	0.067	0.194
681	0.000	-0.007	-0.002	0.055	0.166
682	0.000	-0.007	-0.002	0.046	0.142
683	0.000	-0.007	-0.002	0.038	0.122
684	0.000	-0.008	-0.003	0.031	0.102
685	0.000	-0.007	-0.003	0.026	0.087
686	0.000	-0.006	-0.003	0.022	0.073
687	0.000	-0.006	-0.002	0.019	0.061
688	0.000	-0.006	-0.002	0.017	0.050
689	0.000	-0.007	-0.002	0.012	0.043
690	0.000	-0.006	-0.002	0.010	0.036
691	0.000	-0.006	-0.002	0.009	0.032
692	0.000	-0.005	-0.002	0.006	0.027
693	0.000	-0.005	-0.002	0.005	0.023
694	0.000	-0.005	-0.002	0.004	0.020
695	0.000	-0.005	-0.002	0.003	0.018
696	0.000	-0.005	-0.002	0.003	0.016
697	0.000	-0.005	-0.002	0.002	0.014
698	0.000	-0.004	-0.001	0.002	0.013
699	0.000	-0.004	-0.001	0.002	0.012
700	0.000	-0.004	-0.002	0.001	0.010
701	0.000	-0.005	-0.002	0.001	0.008
702	0.000	-0.005	-0.002	0.001	0.007
703	0.000	-0.005	-0.002	0.001	0.007
704	0.000	-0.005	-0.002	0.000	0.007
705	0.000	-0.004	-0.002	0.000	0.005
706	0.000	-0.005	-0.002	0.000	0.004
707	0.000	-0.006	-0.002	0.000	0.003
708	0.000	-0.007	-0.002	0.000	0.003
709	0.000	-0.007	-0.002	0.000	0.002
710	0.000	-0.006	-0.002	0.000	0.002
711	0.000	-0.005	-0.002	0.000	0.002
712	0.000	-0.005	-0.002	0.000	0.001
713	0.000	-0.006	-0.003	0.000	0.001
714	0.000	-0.006	-0.003	0.000	0.001
715	0.000	-0.006	-0.003	0.000	0.002
716	0.000	-0.006	-0.002	0.000	0.002
717	0.000	-0.006	-0.002	0.000	0.001
718	0.000	-0.007	-0.002	0.000	0.001
719	0.000	-0.006	-0.002	0.000	0.001
720	0.000	-0.006	-0.002	0.000	0.001
721	0.000	-0.005	-0.002	0.000	0.001
722	0.000	-0.004	-0.002	0.000	0.001
723	0.000	-0.004	-0.001	0.000	0.001
724	0.000	-0.005	-0.002	0.000	0.001
725	0.000	-0.007	-0.002	-0.001	0.001
726	0.000	-0.006	-0.003	0.000	0.001
727	0.000	-0.005	-0.003	0.000	0.001
728	0.000	-0.004	-0.002	0.000	0.001
729	0.000	-0.004	-0.001	0.000	0.001
730	0.000	-0.005	-0.001	0.000	0.001
731	0.000	-0.004	-0.001	0.000	0.001
732	0.000	-0.004	-0.001	0.000	0.002

733	0.000	-0.004	-0.002	0.000	0.000
734	0.000	-0.005	-0.003	0.000	0.000
735	0.000	-0.004	-0.002	0.000	0.001
736	0.000	-0.004	-0.002	0.000	0.001
737	0.000	-0.006	-0.002	0.000	0.000
738	0.000	-0.005	-0.002	0.000	0.000
739	0.000	-0.005	-0.002	0.000	0.000
740	0.000	-0.006	-0.003	0.000	0.000
741	0.000	-0.006	-0.003	-0.001	0.000
742	0.000	-0.006	-0.002	-0.001	0.000
743	0.000	-0.007	-0.002	0.000	0.000
744	0.000	-0.009	-0.003	-0.001	0.000
745	0.000	-0.008	-0.003	-0.002	0.000
746	0.000	-0.008	-0.004	-0.002	0.000
747	0.000	-0.007	-0.003	-0.001	0.000
748	0.000	-0.007	-0.003	-0.001	0.000
749	0.000	-0.006	-0.004	-0.001	0.000
750	0.000	-0.007	-0.004	0.000	0.000
751	0.000	-0.008	-0.004	0.000	0.000
752	0.000	-0.008	-0.004	-0.001	0.000
753	0.000	-0.006	-0.001	-0.001	0.001
754	0.000	-0.004	-0.001	-0.001	0.002
755	0.000	-0.003	-0.002	0.000	0.001
756	0.000	-0.005	-0.002	0.000	0.000
757	0.000	-0.004	-0.001	0.000	0.000
758	0.000	-0.003	0.000	0.000	0.000
759	0.000	-0.002	0.000	0.000	0.000
760	0.000	-0.003	-0.001	0.000	0.000
761	0.000	-0.003	-0.001	-0.001	0.000
762	0.000	-0.004	-0.001	-0.001	0.000
763	0.000	-0.006	-0.002	-0.002	0.000
764	0.000	-0.007	-0.003	-0.001	0.000
765	0.000	-0.006	-0.002	-0.001	0.000
766	0.000	-0.005	-0.002	-0.002	0.000
767	0.000	-0.004	-0.001	-0.001	0.000
768	0.000	-0.003	0.000	-0.001	0.000
769	0.000	-0.004	-0.001	-0.001	0.000
770	0.000	-0.003	-0.001	0.000	0.002
771	0.000	-0.005	-0.002	-0.001	0.001
772	0.000	-0.008	-0.002	0.000	0.000
773	0.000	-0.007	-0.001	-0.001	0.000
774	0.000	-0.005	-0.002	-0.002	0.000
775	0.000	-0.005	-0.004	-0.002	0.000
776	0.000	-0.005	-0.002	0.000	0.001
777	0.000	-0.006	0.000	0.000	0.001
778	0.000	-0.004	-0.001	0.000	0.001
779	0.000	-0.004	0.000	0.000	0.001
780	0.000	-0.004	0.000	0.000	0.000
781	0.000	-0.005	-0.001	0.000	0.000
782	0.000	-0.005	-0.001	0.001	0.001
783	0.000	-0.002	-0.001	0.000	0.002
784	0.000	-0.002	-0.001	0.000	0.002
785	0.000	-0.003	-0.002	0.000	0.001
786	0.000	-0.004	-0.002	0.001	0.000
787	0.000	-0.004	-0.002	0.002	0.000
788	0.000	-0.003	-0.001	0.002	0.000

789	0.000	-0.004	-0.001	0.001	0.000
790	0.000	-0.003	0.000	0.000	0.000
791	0.000	-0.001	-0.001	0.000	0.001
792	0.000	-0.002	-0.003	0.000	0.000
793	0.000	-0.004	-0.003	-0.001	0.000
794	0.000	-0.005	-0.002	0.000	0.001
795	0.000	-0.006	-0.003	0.000	0.003
796	0.000	-0.004	-0.002	0.000	0.002
797	0.000	-0.004	-0.001	0.000	0.000
798	0.000	-0.003	-0.001	0.000	0.000
799	0.000	-0.005	-0.005	-0.001	0.000
800	0.000	-0.004	-0.004	-0.001	0.000

YF	RF
S. cheesmaniae LA0421	*S. lycopersicum* cv. Heinz
005	007
no	**4 times**
194,5	329.2
1	1
0.751	0.222
0.736	0.221
0.721	0.220
0.706	0.220
0.692	0.219
0.679	0.219
0.666	0.219
0.653	0.220
0.641	0.221
0.628	0.222
0.617	0.223
0.607	0.223
0.599	0.223
0.592	0.223
0.586	0.225
0.579	0.228
0.574	0.232
0.568	0.236
0.562	0.242
0.555	0.248
0.550	0.255
0.546	0.262
0.543	0.271
0.541	0.278
0.539	0.284
0.534	0.290
0.528	0.297
0.520	0.304
0.511	0.312
0.504	0.321
0.498	0.333
0.493	0.347
0.488	0.360
0.483	0.370
0.476	0.376

Phytoene absorption maximum - at 285 nm; I
maximum - at 331, 347 and 365 nm

Sample #	Sample ID	**Hexane**
**001**		

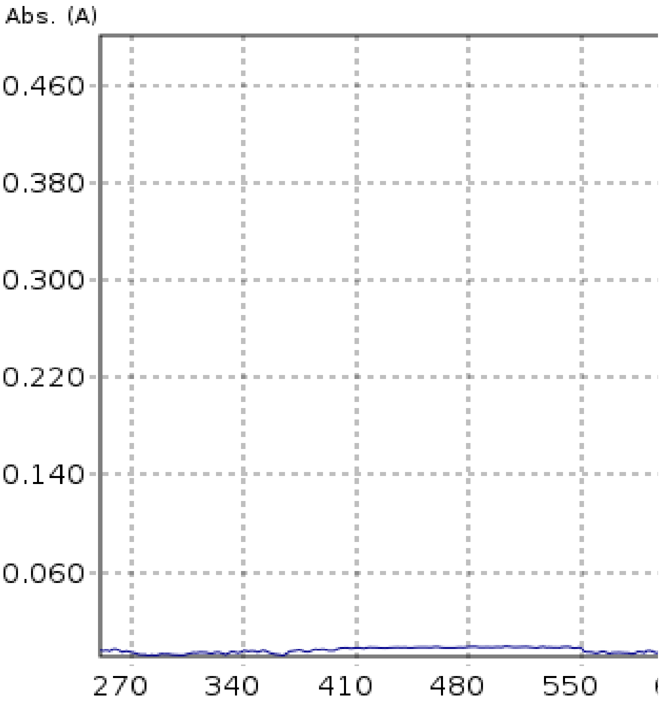

Sample #	Sample ID	**Hexane**
**002**		

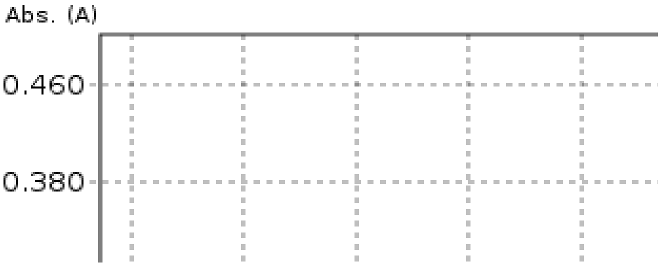

0.467	0.380
0.458	0.382
0.449	0.382
0.441	0.382
0.433	0.383
0.426	0.386
0.419	0.392
0.413	0.401
0.408	0.412
0.403	0.423
0.399	0.432
0.395	0.436
0.392	0.434
0.390	0.426
0.388	0.411
0.386	0.390
0.384	0.364
0.382	0.336
0.379	0.307
0.377	0.277
0.374	0.249
0.370	0.222
0.366	0.198
0.364	0.177
0.362	0.159
0.361	0.145
0.361	0.133
0.361	0.124
0.361	0.118
0.362	0.114
0.364	0.110
0.365	0.108
0.367	0.106
0.369	0.104
0.372	0.103
0.375	0.103
0.378	0.103
0.382	0.103
0.385	0.104
0.389	0.106
0.392	0.108
0.396	0.110
0.400	0.112
0.405	0.114
0.409	0.117
0.413	0.120
0.416	0.123
0.420	0.124
0.423	0.125
0.426	0.126
0.430	0.127
0.433	0.127
0.437	0.127
0.440	0.128
0.445	0.130
0.449	0.133

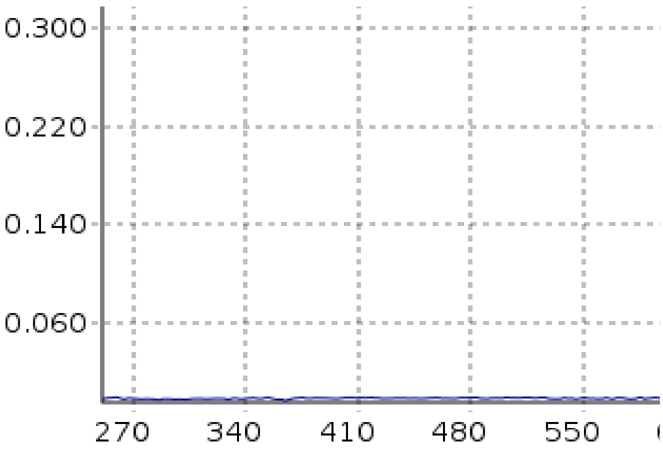

Sample #	Sample ID	
**003**		***S. habrochaites* LA 2144**

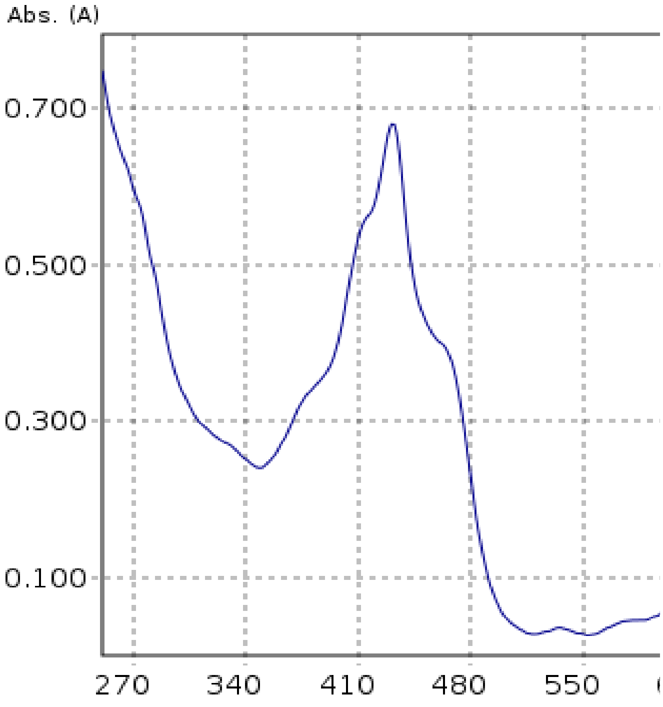

Sample #	Sample ID	
**004**		***S. pennellii* LA0716**

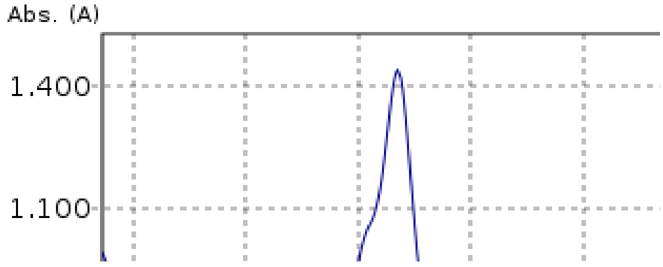

0.454	0.137
0.459	0.141
0.465	0.147
0.471	0.152
0.478	0.156
0.484	0.160
0.489	0.163
0.495	0.166
0.502	0.168
0.509	0.169
0.516	0.168
0.524	0.167
0.531	0.166
0.538	0.165
0.544	0.164
0.550	0.164
0.556	0.165
0.562	0.167
0.568	0.169
0.573	0.173
0.579	0.176
0.584	0.180
0.588	0.183
0.592	0.186
0.596	0.188
0.600	0.190
0.603	0.191
0.606	0.190
0.608	0.188
0.610	0.186
0.611	0.183
0.611	0.180
0.610	0.176
0.609	0.173
0.608	0.170
0.607	0.169
0.604	0.167
0.602	0.166
0.598	0.166
0.595	0.166
0.591	0.168
0.587	0.170
0.583	0.173
0.578	0.176
0.573	0.181
0.569	0.185
0.564	0.190
0.559	0.196
0.554	0.202
0.549	0.207
0.543	0.214
0.537	0.220
0.531	0.227
0.524	0.233
0.518	0.238
0.511	0.244

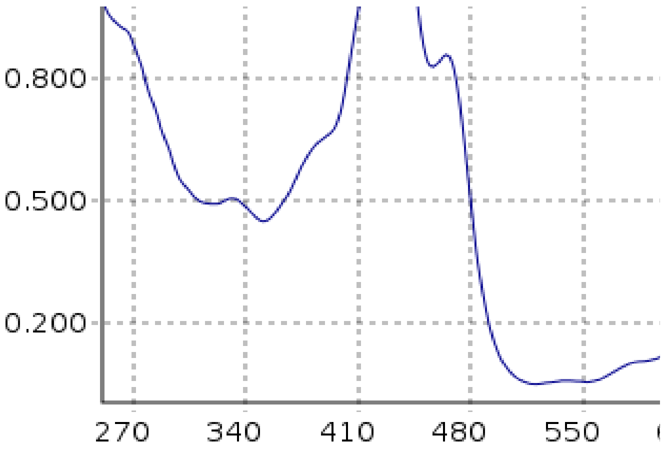

Sample #	Sample ID	
**005**		***S. cheesmaniae* LA0421**

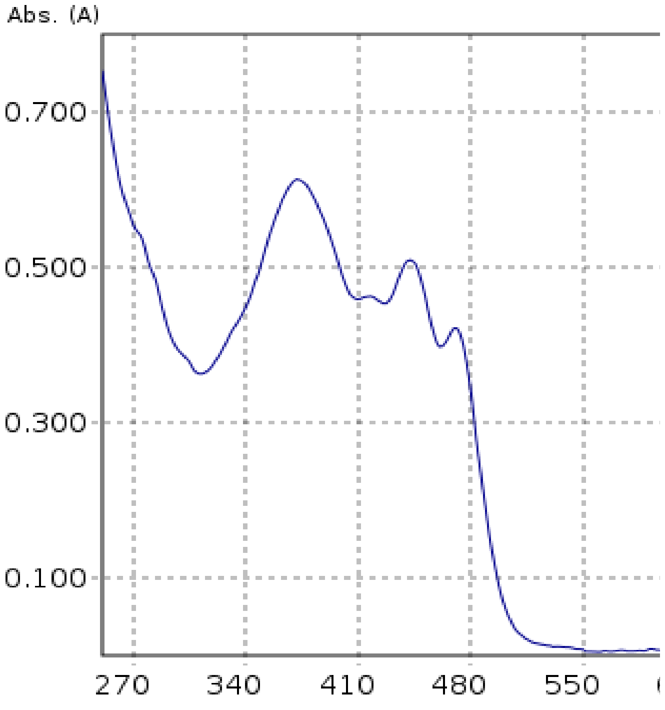

Sample #	Sample ID	
**007**		***S. lycopersicum* cv. Heinz**

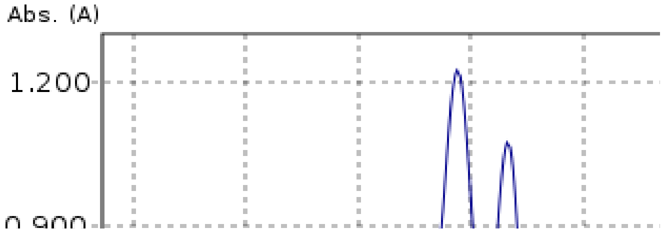

0.505	0.249
0.497	0.254
0.490	0.260
0.484	0.265
0.479	0.271
0.474	0.277
0.469	0.284
0.465	0.291
0.461	0.299
0.459	0.308
0.458	0.318
0.457	0.330
0.457	0.342
0.457	0.356
0.458	0.370
0.459	0.384
0.459	0.398
0.459	0.412
0.460	0.426
0.461	0.439
0.461	0.451
0.460	0.461
0.459	0.470
0.458	0.476
0.456	0.482
0.455	0.487
0.454	0.491
0.452	0.494
0.451	0.499
0.451	0.504
0.451	0.511
0.453	0.520
0.456	0.533
0.459	0.548
0.464	0.566
0.469	0.588
0.474	0.612
0.480	0.639
0.486	0.667
0.491	0.696
0.496	0.724
0.500	0.751
0.504	0.776
0.506	0.798
0.508	0.816
0.508	0.829
0.507	0.838
0.505	0.843
0.503	0.843
0.498	0.840
0.493	0.834
0.486	0.824
0.478	0.813
0.470	0.801
0.462	0.789
0.453	0.779

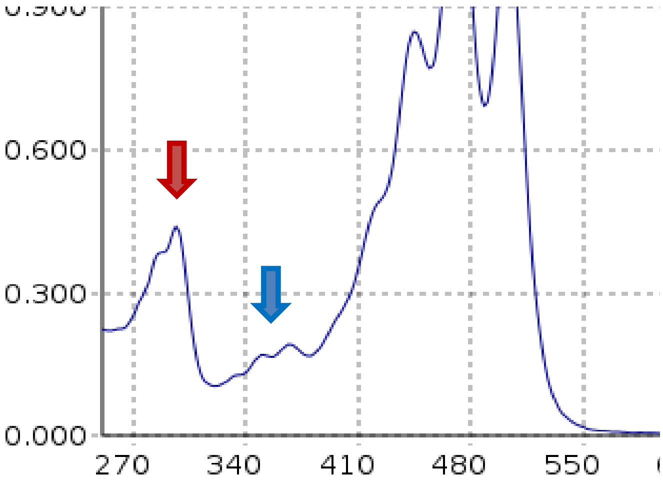

Phytoene absorption maximum - at 285 r
absorption maximum - at 331, 347 and 3(

0.443	0.771
0.433	0.767
0.424	0.767
0.417	0.772
0.409	0.784
0.403	0.803
0.399	0.827
0.396	0.855
0.395	0.888
0.396	0.925
0.398	0.967
0.402	1.012
0.405	1.058
0.409	1.102
0.413	1.141
0.417	1.173
0.419	1.197
0.420	1.214
0.419	1.221
0.416	1.220
0.411	1.209
0.405	1.187
0.396	1.157
0.386	1.120
0.374	1.078
0.362	1.031
0.347	0.983
0.331	0.932
0.313	0.881
0.294	0.832
0.276	0.789
0.260	0.755
0.244	0.729
0.229	0.709
0.214	0.695
0.199	0.688
0.184	0.689
0.169	0.696
0.155	0.713
0.142	0.735
0.130	0.762
0.119	0.795
0.108	0.834
0.098	0.879
0.089	0.924
0.080	0.967
0.072	1.005
0.065	1.036
0.059	1.057
0.054	1.071
0.049	1.072
0.044	1.061
0.040	1.040
0.036	1.007
0.032	0.965
0.030	0.915

0.028	0.859
0.026	0.800
0.024	0.740
0.022	0.679
0.020	0.619
0.019	0.561
0.018	0.507
0.017	0.455
0.016	0.406
0.015	0.362
0.014	0.323
0.013	0.290
0.013	0.259
0.013	0.231
0.012	0.206
0.012	0.182
0.012	0.161
0.011	0.143
0.011	0.126
0.010	0.112
0.010	0.100
0.010	0.090
0.009	0.081
0.009	0.074
0.010	0.066
0.009	0.059
0.009	0.054
0.009	0.049
0.009	0.045
0.009	0.041
0.009	0.038
0.008	0.035
0.009	0.033
0.009	0.030
0.008	0.027
0.007	0.025
0.006	0.024
0.006	0.022
0.006	0.021
0.006	0.020
0.006	0.020
0.005	0.018
0.003	0.015
0.003	0.014
0.003	0.014
0.003	0.013
0.003	0.012
0.003	0.012
0.003	0.011
0.003	0.011
0.002	0.010
0.003	0.010
0.003	0.010
0.003	0.010
0.004	0.010
0.004	0.009

0.003	0.010
0.003	0.009
0.003	0.008
0.003	0.008
0.003	0.008
0.004	0.008
0.004	0.008
0.004	0.008
0.005	0.007
0.005	0.007
0.004	0.007
0.004	0.007
0.003	0.006
0.003	0.006
0.003	0.005
0.004	0.005
0.003	0.005
0.004	0.005
0.004	0.006
0.004	0.006
0.004	0.006
0.003	0.006
0.003	0.005
0.004	0.005
0.005	0.005
0.006	0.005
0.007	0.005
0.007	0.005
0.006	0.005
0.006	0.005
0.005	0.005
0.005	0.005
0.005	0.005
0.005	0.004
0.004	0.004
0.004	0.003
0.005	0.004
0.006	0.005
0.007	0.006
0.007	0.006
0.007	0.004
0.006	0.003
0.006	0.003
0.007	0.003
0.007	0.003
0.007	0.003
0.008	0.004
0.008	0.004
0.009	0.003
0.008	0.003
0.007	0.002
0.008	0.002
0.008	0.003
0.009	0.003
0.009	0.003
0.010	0.003

0.010	0.003
0.010	0.003
0.010	0.003
0.009	0.003
0.008	0.003
0.007	0.003
0.007	0.002
0.007	0.002
0.008	0.003
0.008	0.003
0.008	0.003
0.008	0.002
0.007	0.002
0.008	0.003
0.008	0.003
0.008	0.002
0.008	0.002
0.010	0.002
0.011	0.002
0.011	0.003
0.011	0.003
0.013	0.003
0.013	0.002
0.014	0.002
0.015	0.002
0.015	0.003
0.016	0.003
0.018	0.003
0.018	0.002
0.019	0.002
0.020	0.003
0.022	0.004
0.024	0.003
0.026	0.003
0.027	0.003
0.030	0.004
0.032	0.004
0.035	0.005
0.036	0.004
0.038	0.004
0.038	0.002
0.038	0.002
0.039	0.003
0.040	0.003
0.041	0.003
0.041	0.004
0.039	0.003
0.039	0.002
0.037	0.002
0.034	0.003
0.031	0.003
0.029	0.003
0.026	0.003
0.024	0.002
0.022	0.001
0.020	0.001

0.019	0.002
0.017	0.003
0.015	0.003
0.012	0.002
0.011	0.002
0.010	0.002
0.009	0.002
0.006	0.001
0.004	0.002
0.003	0.002
0.003	0.002
0.002	0.001
0.002	0.002
0.001	0.002
0.000	0.002
0.000	0.002
0.000	0.002
0.000	0.002
0.000	0.002
0.000	0.002
0.000	0.002
0.000	0.004
0.000	0.003
0.000	0.002
0.000	0.002
0.000	0.002
0.000	0.002
0.000	0.002
0.000	0.002
0.000	0.001
-0.001	0.001
0.000	0.001
0.000	0.001
0.000	0.002
0.000	0.003
0.000	0.003
-0.001	0.002
-0.001	0.002
-0.001	0.001
-0.001	0.001
0.000	0.001
0.000	0.002
0.000	0.002
0.000	0.001
0.000	0.001
0.000	0.002
0.000	0.002
0.000	0.002
0.000	0.002
0.000	0.001
0.000	0.001
0.000	0.002
0.000	0.003
-0.001	0.002
0.000	0.003
0.000	0.002

0.000	0.002
0.000	0.001
0.000	0.002
0.000	0.003
-0.001	0.002
-0.001	0.003
-0.001	0.002
-0.002	0.001
-0.001	0.001
-0.002	0.002
-0.001	0.002
-0.001	0.000
-0.001	0.000
-0.001	0.000
-0.001	0.001
-0.001	0.001
-0.001	0.001
0.000	0.001
-0.001	0.000
-0.002	0.000
-0.001	0.001
-0.001	0.001
-0.001	0.002
-0.001	0.001
0.000	0.002
0.000	0.002
0.000	0.002
0.000	0.001
-0.001	0.001
-0.001	0.001
-0.001	0.003
-0.001	0.002
-0.001	0.001
-0.002	0.000
-0.002	0.002
-0.002	0.002
-0.001	0.002
0.000	0.003
-0.001	0.001
-0.002	0.001
-0.002	0.001
-0.002	0.001
0.000	0.000
0.000	0.001
0.000	0.002
0.000	0.002
0.000	0.004
0.000	0.003
0.000	0.002
-0.001	0.002
0.000	0.002
0.000	0.002
0.000	0.002
0.000	0.000
-0.001	0.001
0.000	0.003

-0.001	0.002
-0.002	0.001
-0.002	0.003
-0.001	0.004
-0.002	0.004
-0.001	0.003
0.000	0.006
0.000	0.007
0.000	0.002
0.000	0.002
0.000	0.002
0.000	0.003

Phytofluene absorption

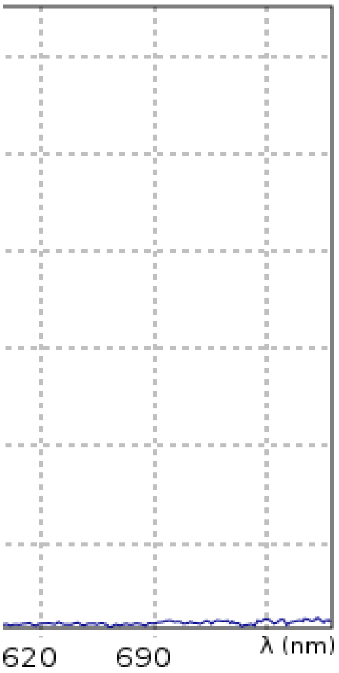

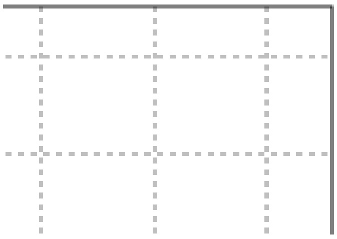

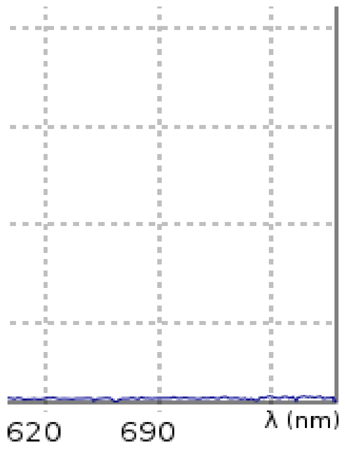
620
690
λ (nm)

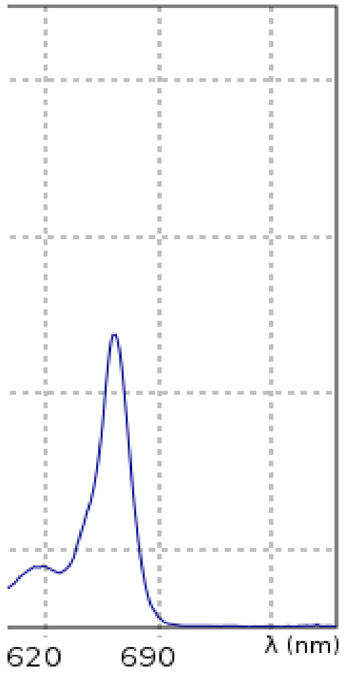
620
690
λ (nm)

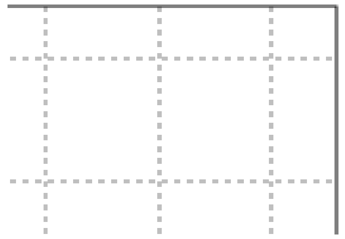

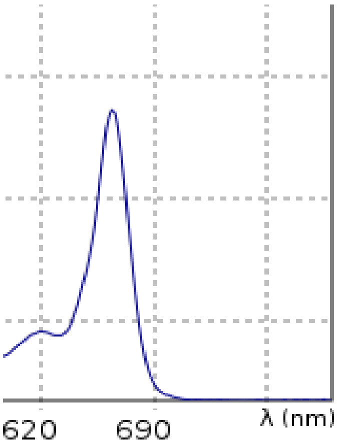
620
690
λ (nm)

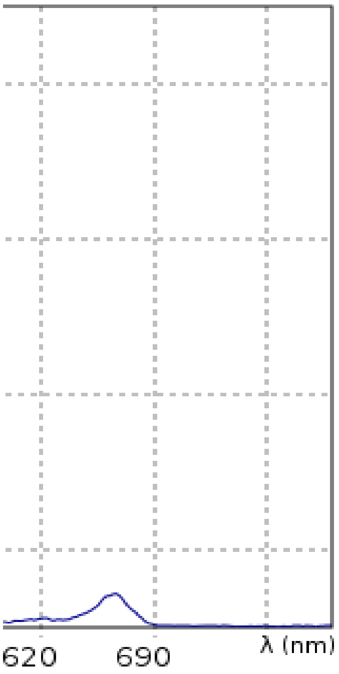
620
690
λ (nm)

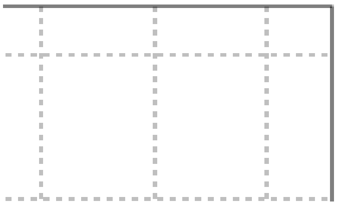

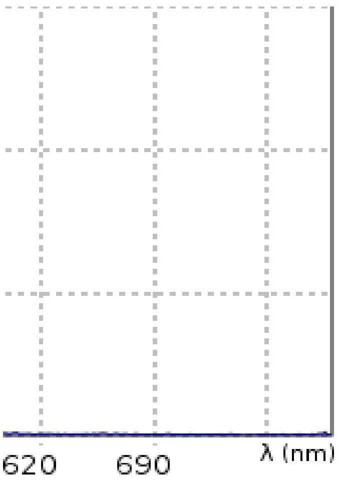

ım; Phytofluene
65 nm
